# Cortical microstructure is associated with disease severity and clinical progression in genetic frontotemporal dementia: a GENFI study

**DOI:** 10.1038/s41380-025-03280-x

**Published:** 2025-10-09

**Authors:** Elena Rodriguez-Vieitez, Melissa T. Rydell, Abbe Ullgren, Victor Montal, Ignacio Illán-Gala, Juan Fortea, Vesna Jelic, Arabella Bouzigues, Lucy L. Russell, Phoebe H. Foster, Eve Ferry-Bolder, John C. van Swieten, Lize C. Jiskoot, Harro Seelaar, Raquel Sanchez-Valle, Robert Laforce, Daniela Galimberti, Rik Vandenberghe, Alexandre de Mendonça, Pietro Tiraboschi, Isabel Santana, Alexander Gerhard, Johannes Levin, Sandro Sorbi, Markus Otto, Florence Pasquier, Simon Ducharme, Chris R. Butler, Isabelle Le Ber, Elizabeth Finger, Maria Carmela Tartaglia, Mario Masellis, James B. Rowe, Matthis Synofzik, Fermin Moreno, Barbara Borroni, Jonathan D. Rohrer, Eric Westman, Caroline Graff

**Affiliations:** 1 Department of Neurobiology, Care Sciences and Society, Division of Neurogeriatrics, Center for Alzheimer Research, Karolinska Institutet, Stockholm, Sweden; 2 Unit for Hereditary Dementias, Theme Inflammation and Aging, Karolinska University Hospital, Stockholm, Sweden; 3 Barcelona Supercomputing Center, Barcelona, Spain; 4 Sant Pau Memory Unit, Neurology Department, Institut d’Investigacions Biomèdiques Sant Pau, Hospital de la Santa Creu i Sant Pau, Universitat Autònoma de Barcelona, Barcelona, Spain; 5 CIBERNED, Network Center for Biomedical Research in Neurodegenerative Diseases, National Institute of Health Carlos III, Madrid, Spain; 6 Department of Neurobiology, Care Sciences and Society, Division of Clinical Geriatrics, Center for Alzheimer Research, Karolinska Institutet, Stockholm, Sweden; 7 Cognitive Clinic, Theme Inflammation and Aging, Karolinska University Hospital, Stockholm, Sweden; 8 Dementia Research Centre, Department of Neurodegenerative Disease, UCL Queen Square Institute of Neurology, London, UK; 9 Sorbonne Université, Paris Brain Institute, Institut du Cerveau – ICM, Inserm U1127, Pitié-Salpêtrière, Paris, France; 10 Department of Neurology, Erasmus Medical Centre, Rotterdam, Netherlands; 11 Alzheimer’s disease and Other Cognitive Disorders Unit, Neurology Service, Hospital Clínic de Barcelona, Institut d’Investigacións Biomèdiques August Pi I Sunyer, University of Barcelona, Barcelona, Spain; 12 Clinique Interdisciplinaire de Mémoire, Département des Sciences Neurologiques, CHU de Québec, and Faculté de Médecine, Université Laval, Québec City, QC Canada; 13 Fondazione Ca’ Granda, IRCCS Ospedale Maggiore Policlinico, Milan, Italy; 14 Dept. of Biomedical, Surgical and Dental Sciences, University of Milan, Milan, Italy; 15 Laboratory for Cognitive Neurology, Department of Neurosciences, KU Leuven, Leuven, Belgium; 16 Neurology Service, University Hospitals Leuven, Leuven, Belgium; 17 Leuven Brain Institute, KU Leuven, Leuven, Belgium; 18 Faculty of Medicine, University of Lisbon, Lisbon, Portugal; 19 Division of Neurology, Fondazione IRCCS Istituto Neurologico Carlo Besta, Milan, Italy; 20 Faculty of Medicine, University of Coimbra, Coimbra, Portugal; 21 Center for Innovative Biomedicine and Biotechnology, University of Coimbra, Coimbra, Portugal; 22 Division of Psychology Communication and Human Neuroscience, Wolfson Molecular Imaging Centre, University of Manchester, Manchester, UK; 23 Department of Geriatric Medicine, Klinikum Hochsauerland GmbH, Arnsberg, Germany; 24 Department of Nuclear Medicine, Center for Translational Neuro- and Behavioral Sciences, University Hospital Essen, Essen, Germany; 25 Department of Neurology, Ludwig-Maximilians-University, Munich, Germany; 26 Munich Cluster of Systems Neurology (SyNergy), Munich, Germany; 27 German Center for Neurodegenerative Diseases (DZNE), site Munich, Germany; 28 Department of Neurofarba, University of Florence, Florence, Italy; 29 Fondazione Don Carlo Gnocchi, Istituto di Ricovero e Cura a Carattere Scientifico, Florence, Italy; 30 Department of Neurology, Martin-Luther University Halle-Wittenberg, Halle (Saale), Germany; 31 University of Lille and INSERM 1172, Lille, France; 32 Department of Psychiatry, Douglas Mental Health University Institute, McGill University, Montreal, QC Canada; 33 McConnell Brain Imaging Centre, Montreal Neurological Institute, McGill University, Montreal, QC Canada; 34 Department of Brain Sciences, Imperial College London, London, UK; 35 The George Institute for Global Health, Imperial College London, London, UK; 36 Department of Clinical Neurological Sciences, University of Western Ontario, London, ON Canada; 37 Tanz Centre for Research in Neurodegenerative Diseases, University of Toronto, Toronto, ON Canada; 38 Sunnybrook Health Sciences Centre, Sunnybrook Research Institute, University of Toronto, Toronto, ON Canada; 39 Department of Clinical Neurosciences, University of Cambridge, Cambridge, UK; 40 Cambridge University Hospitals NHS Trust, University of Cambridge, Cambridge, UK; 41 Medical Research Council Cognition and Brain Sciences Unit, University of Cambridge, Cambridge, UK; 42 Department of Neurodegenerative Diseases, Hertie-Institute for Clinical Brain Research and Center of Neurology, University of Tübingen, Tübingen, Germany; 43 Center for Neurodegenerative Diseases (DZNE), Tübingen, Germany; 44 Cognitive Disorders Unit, Department of Neurology, Donostia University Hospital, San Sebastian, Gipuzkoa Spain; 45 Neuroscience Area, Biodonostia Health Research Institute, San Sebastian, Gipuzkoa Spain; 46 Center for Biomedical Research in Neurodegenerative Disease (CIBERNED), Carlos III Health Institute, Madrid, Spain; 47 Department of Clinical and Experimental Sciences, University of Brescia, Brescia, Italy; 48 Molecular Markers Laboratory, IRCCS Istituto Centro San Giovanni di Dio Fatebenefratelli, Brescia, Italy

**Keywords:** Prognostic markers, Diseases, Psychology, Genetics, Neuroscience

## Abstract

The study of genetic frontotemporal dementia (FTD) allows investigating its earliest presymptomatic stages. Using cross-sectional T1-weighted and diffusion-weighted MRI, we test the hypothesis that cortical microstructural alterations, quantified as cortical mean diffusivity (cMD), are detectable earlier and are more strongly associated with clinical progression than cortical thickness (CTh). The sample comprised *n* = 710 individuals (47.8 ± 13.5 years, 56.6% female, 14.1 ± 3.3 years of education), including 118 symptomatic carriers and 305 presymptomatic carriers with mutations in *C9orf72*, *GRN* or *MAPT* genes, and 287 non-carriers, collected from 24 GENFI sites. A subset of *n* = 453 individuals (289 carriers, 164 non-carriers) were investigated across Clinical Dementia Rating (CDR) = 0, 0.5 and ≥1 stages. Two subsets had longitudinal clinical outcome measures, including *n* = 403 individuals (239 carriers, 164 non-carriers) with Cambridge Behavioural Inventory-Revised scores during 2.8 ± 1.6 years, and *n* = 261 individuals (164 carriers, 97 non-carriers) with CDR Sum-of-Boxes scores during 2.0 ± 0.8 years. Regional cMD and CTh were entered into linear mixed-effects models incorporating age, sex and education as covariates; site, and individual nested within site were random intercepts. The results demonstrated that cMD is more sensitive than CTh to track early cortical injury, with elevated cMD first observed at CDR = 0 in *C9orf72* carriers, followed by *MAPT* carriers (from CDR = 0.5 stage), and by *GRN* carriers (beginning at CDR ≥ 1). At all stages, cortical microstructural injury had stronger effect size and was more widespread than cortical thinning. In all mutation carrier types, cMD was more strongly associated than CTh with subsequent clinical progression. Cortical microstructure is a promising biomarker to identify at-risk individuals before atrophy and clinical progression, with utility in therapeutic trials.

## Introduction

The socio-economic burden of neurodegenerative diseases is of growing concern due to global population ageing and the scarcity of effective disease-modifying therapies [1]. Among them, frontotemporal dementia (FTD) encompasses a heterogeneous spectrum of neurodegenerative diseases with overlapping patterns of clinical presentation, histopathological features and genetic factors. FTD mainly causes behavioural changes (behavioural variant FTD [bvFTD]) or language impairment (primary progressive aphasia [PPA]) [2]. About one third of all FTD is genetic, with an autosomal-dominant inheritance. The three most common disease-causing mutations are located in chromosome 9 open reading frame 72 (*C9orf72*), progranulin (*GRN*) and microtubule-associated protein tau (*MAPT*), respectively. Neuropathologically, mutations in the *C9orf72* or *GRN* genes are associated with the accumulation of misfolded TDP-43 protein in the brain, while mutations in the *MAPT* gene are characterized by deposits of neurofibrillary tau tangles [3, 4]. In genetic FTD, mutation carriers develop the disease with high penetrance, although with some variability in age of symptoms onset [5]. Despite these uncertainties, the study of genetic FTD allows one to investigate the earliest stages of pathological changes in the brain, years prior to the development of symptoms.

Neuroimaging techniques are increasingly used for differential diagnosis in the spectrum of FTD and to track disease progression. In particular, MRI is an established instrument to detect macrostructural neurodegenerative changes such as cortical atrophy quantified by cortical thickness (CTh). The characteristic patterns of cortical atrophy are included in the diagnostic criteria of probable bvFTD [6], and in image-supported clinical diagnoses of nonfluent-variant PPA (nfvPPA) and logopenic-variant PPA (lvPPA) [7]. However, standard structural MRI techniques have limited sensitivity to brain abnormalities in early stages of the disease [8], and differential clinical diagnosis remains a complex challenge. With the advent of new preventive and therapeutic trials for FTD, there is a pressing need for early prognostic biomarkers that can predict short-term disease progression, for selection of at-risk individuals for enrollment in trials, and as outcome measures of therapeutic efficacy [2].

FTD has a long presymptomatic and prodromal phase. Several studies in genetic FTD have reported incipient cortical atrophy 10–20 years prior to symptoms onset [9]. Structural alterations are earliest for the *C9orf72* mutation carriers [10], suggesting the need to stratify neuroimaging analyses by mutation type (*C9orf72, GRN* and *MAPT*). In addition to structural MRI, the acquisition of diffusion-weighted MRI sequences allows to obtain microstructural information by tracking the displacement of water molecules within the brain [11, 12]. While diffusion-weighted imaging is typically evaluated in white matter, novel quantification methods allow to measure cortical mean diffusivity (cMD), the average diffusion of water molecules along any possible direction, within the grey matter [13, 14], as a measure of cortical injury due to, for example, disruption of cellular membranes and synaptic damage at the microstructural level. A reduction of density within cortical grey matter would result in an increase of cMD, as water molecules move more freely. Increased cMD has been reported in the Alzheimer continuum [14–16] and in sporadic FTD [17]. In sporadic bvFTD, areas of elevated cMD in the brain were more widespread and had greater discriminative ability between FTD patients and controls than areas of reduced CTh [17]. Recent studies on cMD in PPA and in the amyotrophic lateral sclerosis (ALS)-FTD spectrum showed increased cMD in areas of cortical atrophy but also extending beyond non-atrophic areas [18, 19]. It has also been reported that cMD increases when approaching the estimated age of clinical onset in autosomal-dominant Alzheimer disease [20]. A previous study showed elevated cMD in tau-related areas in preclinical Alzheimer disease, suggesting that increased cMD might be a proxy for tau accumulation [21]. Furthermore, elevated cMD at baseline predicted faster rates of subsequent cognitive decline, hippocampal atrophy [21] and tau accumulation [22]. Altogether, these findings suggest cMD to be a sensitive biomarker for the identification of at-risk individuals, prior to widespread atrophy, with potential utility as a sensitive biomarker across neurodegenerative diseases including FTD.

In this multicentre study, we aimed to assess mutation-specific cortical microstructure in comparison with macrostructure by analysing two MRI acquisition modalities: T1-weighted and diffusion-weighted MRI data in a cross-sectional sample of genetic FTD mutation carriers and non-carriers. We test the hypothesis that the three different mutation types would have distinct cortical microstructural profiles, and that cMD would increase in mutation carriers as the disease advances. Our central hypothesis is that alterations in cortical microstructure, as measured by cMD, can be detected at earlier disease stages and are more strongly associated with subsequent longitudinal clinical progression than cortical macrostructural alterations measured by CTh. To test this hypothesis, we performed T1-weighted and diffusion-weighted MRI scans in a multicentre international cohort of genetic FTD (GENetic FTD Initiative-GENFI), where all mutation carriers were stratified by mutation type *C9orf72*, *GRN* and *MAPT* with the following aims: (i) assess the association between regional cortical microstructure (cMD) and macrostructure (CTh) respectively with age in mutation carriers compared with non-carriers using an interaction analysis; (ii) assess brain regional patterns of cMD and CTh in genetic FTD cross-sectionally by comparing presymptomatic mutation carriers (pMC) and symptomatic mutation carriers (sMC) respectively vs non-carriers; (iii) assess regional patterns of cMD and CTh at different stages of disease progression as measured by the global Clinical Dementia Rating plus National Alzheimer’s Coordinating Center Behaviour and Language with the addition of neuropsychiatric and motor assessments, also known as the CDR® plus NACC FTLD-NM rating scale (here abbreviated as GENFI-CDR), in mutation carriers compared with non-carriers; (iv) quantify the association between regional and global cMD and CTh at baseline with subsequent longitudinal change in clinical outcome measures including the Cambridge Behavioural Inventory-Revised (CBI-R) and the GENFI-CDR Sum-of-Boxes (GENFI-CDR-SOB).

## Materials and methods

### Study participants and clinical assessment

We selected all available participants from the GENFI multicentre cohort fifth data freeze at their earliest available time point, with the inclusion criteria that they had 3-Tesla MRI scans, with both T1-weighted and diffusion-weighted modalities available at the same time point. These inclusion criteria resulted in a sample of *n* = 773 individuals. After quality control of T1-weighted and diffusion-weighted images, the sample comprised *n* = 710 individuals (47.8 ± 13.5 [range 19.4–86.6] years, 56.6% female, 14.1 ± 3.3 years of education) from 24 unique GENFI sites, including 118 symptomatic mutation carriers (sMC) and 305 presymptomatic carriers (pMC) with mutations in *C9orf72*, *GRN* or *MAPT* genes, and 287 non-carriers serving as controls (Supplementary Table 1). Further inclusion/exclusion criteria and clinical assessment are described in Supplementary Methods.

### Study subsample for disease staging

For staging of disease severity, we used the CDR® plus NACC FTLD-NM rating scale [23]. Here we have abbreviated it as GENFI-CDR and it had values ranging from 0 to 3; higher global GENFI-CDR scores indicate more advanced disease stage.

For cross-sectional analyses of neuroimaging data across stages of disease severity, we investigated a subset of individuals who also had available global GENFI-CDR at baseline, used to classify mutation carriers into three stages of disease severity (GENFI-CDR = 0; 0.5 and ≥1). This subset included *n* = 453 individuals (46.7 ± 13.6 years, 55.2% female, 14.3 ± 3.4 years of education) comprising 289 carriers and 164 non-carriers (Table 1).

**Table 1:** Demographic information of the subset with GENFI Clinical Dementia Rating (GENFI-CDR) at baseline (*n* = 453).

	Non-carriers	*C9orf72* carriers			*GRN* carriers			*MAPT* carriers		
		CDR 0	CDR 0.5	CDR ≥ 1	CDR 0	CDR 0.5	CDR ≥ 1	CDR 0	CDR 0.5	CDR ≥ 1
No. of participants, *n*	164	63	25	40	64	21	28	24	14	10
Age, mean yr (SD)	43.8 (12.5)	42.9 (12.2)	46.7 (12.0)	61.5*** (8.4)	41.7 (12.0)	50.9* (11.7)	62.7*** (7.8)	38.2* (12.5)	44.6 (10.8)	60.6*** (6.4)
Sex, female *n* (% female)	91 (55.5%)	36 (57.1%)	15 (60.0%)	17 (42.5%)	40 (62.5%)	12 (57.1%)	13 (46.4%)	15 (62.5%)	7 (50.0%)	4 (40.0%)
Education, mean yr (SD)	14.7 (3.3)	14.3 (3.3)	14.2 (2.5)	12.7** (3.5)	15.3 (3.6)	13.0 (4.1)	12.3** (3.4)	14.6 (3.0)	14.4 (2.3)	13.8 (2.7)
pMC / sMC	–	63 / 0	21 / 4	2 / 38	64 / 0	18 / 3	1 / 27	24 / 0	10 / 4	2 / 8
GENFI-CDR-SOB mean (SD)	0.3 (0.9)	0.0*** (0.0)	1.5*** (1.0)	11.4*** (6.0)	0.0*** (0.0)	1.2*** (0.9)	10.4*** (6.4)	0.0*** (0.0)	1.4*** (0.9)	7.8*** (4.9)
CBI-R, mean (SD) Missing *n* (%)	4.9 (7.4) 22 (13.4%)	6.5 (8.5) 10 (15.9%)	11.1 (13.2) 5 (20.0%)	67.1*** (29.2) 3 (7.5%)	3.6 (6.8) 6 (9.4%)	11.9* (12.5) 2 (9.5%)	51.0*** (26.2) 2 (7.1%)	6.4 (11.1) 3 (12.5%)	11.8 (14.3) 2 (14.3%)	35.8** (20.9) 1 (10.0%)
MMSE, mean (SD) Missing *n* (%)	29.4 (1.2) 2 (1.2%)	29.1 (1.2) 0 (0.0%)	28.6 (2.1) 0 (0.0%)	24.6*** (4.5) 2 (5.0%)	29.6 (0.7) 2 (3.1%)	28.8 (1.9) 1 (4.8%)	21.0*** (6.9) 1 (3.6%)	29.5 (0.8) 1 (4.2%)	28.6 (2.1) 1 (7.1%)	22.6** (5.7) 1 (10.0%)

The table includes statistical comparisons of demographic data for the comparisons between mutation carriers at different stages of disease severity (GENFI-CDR 0, 0.5 and ≥ 1) vs non-carriers (NC), stratified by gene type *C9orf72*, *GRN* and *MAPT*. *P*-values correspond to pairwise t-tests for continuous variables and χ^2^-tests for categorical variables. *P < 0.05, **P < 0.01, ***P < 0.001.

*CBI-R* Cambridge Behavioural Inventory-Revised; *CDR* Clinical Dementia Rating (same as GENFI-CDR); *GENFI-CDR-SOB* GENFI Clinical Dementia Rating Sum-of-Boxes; *MMSE* Mini-Mental State Examination; *No*. number; *pMC* presymptomatic mutation carrier; *SD* standard deviation; *sMC* symptomatic mutation carrier.

### Study subsamples with longitudinal clinical follow-up data

To investigate the association between baseline multimodal neuroimaging data with subsequent longitudinal clinical evolution, we selected individuals that had at least one baseline and one follow-up clinical outcome assessments, and we performed separate analyses for both CBI-R and GENFI-CDR-SOB longitudinal follow-ups. The CBI-R total score [24–26] was used as a marker of FTD-specific functional and/or behavioural changes. The CBI-R values ranged from 0 to 163 (of max=180); higher values reflect greater impairment. The GENFI-CDR-SOB is an FTD-specific marker of global cognitive performance that includes behaviour, language, neuropsychiatric and motor symptoms, with a greater dynamic range (from 0 to 27) than the global GENFI-CDR values; higher GENFI-CDR-SOB scores indicate greater impairment. In addition, participants were evaluated using Mini-Mental State Examination (MMSE) at baseline, as a marker of global cognitive performance not specific to FTD. MMSE scores ranged from 7 to 27; lower MMSE scores indicate worse performance.

For longitudinal CBI-R, the sample consisted of *n* = 403 individuals (48.4 ± 13.4 years, 59.1% female, 14.4 ± 3.5 years of education, 2.8 ± 1.6 [range 0.8–7.1] years total clinical follow-up, with a range of 2–7 visits) distributed into 239 carriers and 164 non-carriers (Supplementary Table 2). For longitudinal GENFI-CDR-SOB, the sample included *n* = 261 individuals (46.4 ± 13.8 years, 57.9% female, 14.6 ± 3.5 years of education, 2.0 ± 0.8 [range 0.8–4.1] years total clinical follow-up, with a range of 2–4 visits) distributed into 164 carriers and 97 non-carriers (Supplementary Table 2).

### Acquisition of T1-weighted and diffusion-weighted MRI scans

All individuals underwent T1-weighted and diffusion-weighted MRI sequences following GENFI standard image acquisition protocols at scanners from General Electrics, Philips or Siemens, as previously reported [10]. To improve data homogeneity we required that all scanning was performed at 3-Tesla MRI scanners using a sagittal T1-weighted magnetization-prepared rapid gradient echo protocol. With these inclusion criteria, we identified *n* = 773 individuals with a complete set of concurrent 3-Tesla structural and diffusion-weighed MRI scans. After exclusion of *n* = 44 individuals that had image preprocessing errors due to image artifacts and co-registration failures, and the subsequent exclusion of *n* = 19 individuals due to being outliers in their global structural or diffusion-weighted MRI quantitative measures, the final sample consisted of *n* = 710 individuals (Supplementary Table 1) from 24 unique GENFI sites.

### Cortical thickness processing

The 3D T1-weighted MRI images were processed with the TheHiveDB system using FreeSurfer 7.1.1 cross-sectional stream and quality-controlled as previously described [27]. Regional CTh values were extracted from 68 regions-of-interest (ROIs) as defined by the Desikan-Killiany atlas [28] incorporated in FreeSurfer 7.1.1 and used for subsequent analyses.

### Cortical mean diffusivity processing

Processing of diffusion-weighted images was integrated with T1-weighted MRI CTh analysis, using an in-house surface-based diffusion tensor imaging (DTI) approach, which uses tools from the FSL package (v5.0.9) and Freesurfer 7.1.1. Diffusion-weighted images were motion-corrected, skull-stripped and diffusion tensor-fitted. DTI-fitted images were co-registered to each subject’s T1 native space using the *bbregister* tool. Whole-brain mean diffusivity maps were sampled at each cortical vertex in the midpoint between white/pial surfaces, projected onto the subject’s cortical surface space and registered to the Freesurfer standard space. This surface-based approach overcomes limitations of voxel-based methods when used in cortical analyses, where the inclusion of cerebrospinal fluid (CSF) in grey matter voxels can confound the cMD measures. Despite cMD being a protocol-sensitive metric, the number of gradient directions does not strongly impact the cMD metric [14].

### Statistical analysis

All statistical analyses in mutation carriers were performed stratified by mutation type (*C9orf72*, *GRN*, *MAPT*) and all non-carriers were pooled together into one control group, for all Aims 1–4 of the study. Prior to statistical analyses, the distributions of all continuous demographic and neuroimaging variables were graphically inspected using histograms and quantile-quantile plots. Demographic data were compared pairwise between pMC vs NC and sMC vs NC groups using t-tests and χ^2^-tests for continuous and categorical variables, respectively. Two-sided *P*-values below 0.05 were considered significant. After neuroimaging variables cMD and CTh had been both extracted from the same 68 cortical ROIs of the Desikan-Killiany atlas [28], they were z-transformed via mean centring and unit variance scaling prior to further analyses. Similarly, all cognitive data were z-transformed. Outliers (values beyond mean±3 SD range) were excluded.

Linear mixed-effects models (LMEMs) were applied to perform the four aims of the study; the covariance matrix of the residuals was modelled using an unstructured covariance matrix, and models were implemented using restricted maximum likelihood estimation. Statistical significance for all tests was set at 5% (α = 0.05, two-sided). All *P*-values were adjusted for multiple comparisons to account for repeated measures over 68 ROIs, using the Benjamini-Hochberg method for controlling false discovery rates (FDR) (q < 0.05, two-sided) for each mutation type separately.

After implementing the ROI-based statistical models of Aims 1–3 below, the proportion of brain regions with significant findings in cMD, and the respective proportion of brain regions with significant findings in CTh were compared statistically using a χ^2^ test; *P* < 0.05 was considered significant.

All statistical analyses were performed in R (version 4.3.2) using Rstudio environment (version 2023.12.0). LMEMs were implemented using nlme package (version 3.1). Figures were made using ggplot2 and ggseg packages [29].

### Aim 1

To explore whether the associations of regional neuroimaging variables, cMD_ROI_ and CTh_ROI_, with age were significantly different between mutation carriers and non-carriers in the whole cohort, we built LMEMs including an interaction term between fixed-effects factor mutation status (carriers vs non-carriers) and age, also including these factors as independent predictors (Eqs. 1); in all analyses, separate models were built for mutation carriers stratified by mutation type:

1a $${{{{\rm{cMD}}}}}_{{{{\rm{ROI}}}}} \sim 	 {{{{\rm{\beta }}}}}_{0}+{{{{\rm{\beta }}}}}_{1}\,{{{\rm{mutation}}}}\,{{{\rm{status}}}}+{{{{\rm{\beta }}}}}_{2}\,{{{\rm{age}}}}+{{{{\rm{\beta }}}}}_{3}\,{{{\rm{mutation}}}}\,{{{\rm{status}}}}\times {{{\rm{age}}}}\\ 	 + {{{{\rm{\beta }}}}}_{4}\,{{{\rm{sex}}}}+{{{\rm{random}}}}\,{{{\rm{intercept}}}}({{{\rm{GENFI}}}}\,{{{\rm{site}}}})+{{{\rm{\varepsilon }}}}$$

1b $${{{{\rm{CTh}}}}}_{{{{\rm{ROI}}}}} \sim 	 {{{{\rm{\beta }}}}}_{0}+{{{{\rm{\beta }}}}}_{1}\,{{{\rm{mutation}}}}\,{{{\rm{status}}}}+{{{{\rm{\beta }}}}}_{2}\,{{{\rm{age}}}}+{{{{\rm{\beta }}}}}_{3}\,{{{\rm{mutation}}}}\,{{{\rm{status}}}}\times {{{\rm{age}}}}\\ 	 + {{{{\rm{\beta }}}}}_{4}\,{{{\rm{sex}}}}+{{{\rm{random}}}}\,{{{\rm{intercept}}}}({{{\rm{GENFI}}}}\,{{{\rm{site}}}})+{{{\rm{\varepsilon }}}}$$

where mutation status (carrier/non-carrier) is a categorical variable, β_0_ is the intercept and β_1_ to β_4_ are fixed-effects coefficients, with β_3_ representing the coefficient of the interaction term. A random intercept is included to account for the GENFI site where data were collected, and ε is an error term.

### Aim 2

LMEMs were built to perform pairwise comparisons of cMD_ROI_ and of CTh_ROI_ between groups of participants. The dichotomous variable “group” was defined to compare neuroimaging variables between pMC or sMC and NC (group = pMC vs NC, or sMC vs NC) across the whole cohort (Supplementary Table 1). Separate LMEMs were built for each ROI, according to the formulas:

2a $${{{{\rm{cMD}}}}}_{{{{\rm{ROI}}}}} \sim {{{{\rm{\beta }}}}}_{0}+{{{{\rm{\beta }}}}}_{1}\,{{{\rm{group}}}}+{{{{\rm{\beta }}}}}_{2}\,{{{\rm{age}}}}+{{{{\rm{\beta }}}}}_{3}\,{{{\rm{sex}}}}+{{{\rm{random}}}}\,{{{\rm{intercept}}}}({{{\rm{GENFI}}}}\,{{{\rm{site}}}})+{{{\rm{\varepsilon }}}}$$

2b $${{{{\rm{CTh}}}}}_{{{{\rm{ROI}}}}} \sim {{{{\rm{\beta }}}}}_{0}+{{{{\rm{\beta }}}}}_{1}\,{{{\rm{group}}}}+{{{{\rm{\beta }}}}}_{2}\,{{{\rm{age}}}}+{{{{\rm{\beta }}}}}_{3}\,{{{\rm{sex}}}}+{{{\rm{random}}}}\,{{{\rm{intercept}}}}({{{\rm{GENFI}}}}\,{{{\rm{site}}}})+{{{\rm{\varepsilon }}}}$$

where β_0_ is the intercept, and β_1_ to β_3_ are fixed-effects coefficients. A random intercept was added to account for GENFI site, and ε is an error term.

### Aim 3

LMEMs were built to perform pairwise comparisons of cMD_ROI_ and of CTh_ROI_ between groups of participants (Eqs. 2), in the study subsample with GENFI-CDR data available at baseline (Table 1). Here, the dichotomous variable “group” was used to compare carriers, stratified by mutation type, at different stages of disease severity vs non-carriers (GENFI-CDR = 0 mutation carriers vs NC, GENFI-CDR = 0.5 mutation carriers vs NC, and GENFI-CDR ≥ 1 mutation carriers vs NC).

### Aim 4

To test whether baseline cMD_ROI_ and CTh_ROI_ were associated with subsequent longitudinal clinical progression, we built LMEMs (Eqs. 3), that were applied to mutation carriers, with analyses stratified by mutation type. All neuroimaging, clinical outcome variables and continuous covariates (age at baseline and time) had been z-transformed via mean centring and unit variance scaling prior to statistical analyses. Also, we tested the same statistical models within the non-carrier group alone to ascertain that the regional cMD_ROI_ and CTh_ROI_ neuroimaging measures were not associated with subsequent longitudinal clinical changes in this group. Separate models were built for two clinical outcome variables, CBI-R and GENFI-CDR-SOB (Eqs. 3):

3a $${{{\rm{Clinical}}}}\,{{{\rm{outcome}}}}\,{{{\rm{variable}}}},{{{\rm{longit}}}} \sim 	{{{{\rm{\beta }}}}}_{0}+{{{{\rm{\beta }}}}}_{1}\,{{{{\rm{cMD}}}}}_{{{{\rm{ROI}}}}}+{{{{\rm{\beta }}}}}_{2}\,{{{\rm{time}}}}+{{{{\rm{\beta }}}}}_{3}\,{{{{\rm{cMD}}}}}_{{{{\rm{ROI}}}}} \\ 	 \times {{{\rm{time}}}}+{{{{\rm{\beta }}}}}_{4}\,{{{\rm{age}}}}\,{{{\rm{at}}}}\,{{{\rm{baseline}}}}\,+\,{{{{\rm{\beta }}}}}_{5}\,{{{\rm{sex}}}} \\ 	 +{{{{\rm{\beta }}}}}_{6}{{{\rm{education}}}}+{{{\rm{random}}}}\,{{{\rm{intercept}}}}\\ 	 ({{{\rm{GENFI}}}}\,{{{\rm{site}}}}/{{{\rm{individual}}}})+{{{\rm{\varepsilon }}}}$$

3b $${{{\rm{Clinical}}}}\,{{{\rm{outcome}}}}\,{{{\rm{variable}}}},{{{\rm{longit}}}} \sim 	{{{{\rm{\beta }}}}}_{0}+{{{{\rm{\beta }}}}}_{1}\,{{{{\rm{CTh}}}}}_{{{{\rm{ROI}}}}}+{{{{\rm{\beta }}}}}_{2}\,{{{\rm{time}}}}+{{{{\rm{\beta }}}}}_{3}\,{{{{\rm{CTh}}}}}_{{{{\rm{ROI}}}}} \\ 	 \times {{{\rm{time}}}}\,+{{{{\rm{\beta }}}}}_{4}\,{{{\rm{age}}}}\,{{{\rm{at}}}}\,{{{\rm{baseline}}}}\,+\,{{{{\rm{\beta }}}}}_{5}\,{{{\rm{sex}}}} \\ 	 +{{{{\rm{\beta }}}}}_{6}\,{{{\rm{education}}}}+{{{\rm{random}}}}\,{{{\rm{intercept}}}} \\ 	 ({{{\rm{GENFI}}}}\,{{{\rm{site}}}}/{{{\rm{individual}}}})+{{{\rm{\varepsilon }}}}$$

where β_0_ is the intercept and β_1_ to β_6_ are fixed-effects coefficients, with β_3_ representing the coefficient of the interaction term between baseline neuroimaging variables cMD_ROI_ or CTh_ROI_ and time, measured in years from baseline to each of the longitudinal clinical visits. The random intercept accounts for repeated measures in the same individual, nested within each GENFI site, and ε is an error term. Finally, we repeated these LMEM models for global cMD and global CTh measures at baseline, calculated as the average of respective regional neuroimaging variables across the 68 ROIs.

## Results

### Participants characteristics

When comparing demographic data between pMC vs NC and between sMC vs NC, it was noted that sMC were older than NC for all mutation types, and pMC were younger than NC for *C9orf72* and *MAPT* pMC. The *C9orf72* sMC group had greater proportion of males than NC, and the *C9orf72* sMC and *GRN* sMC groups were less educated than NC (Supplementary Table 1).

### Association of cortical microstructure and macrostructure with age in carriers vs non-carriers (Aim 1)

**Fig. 1:**
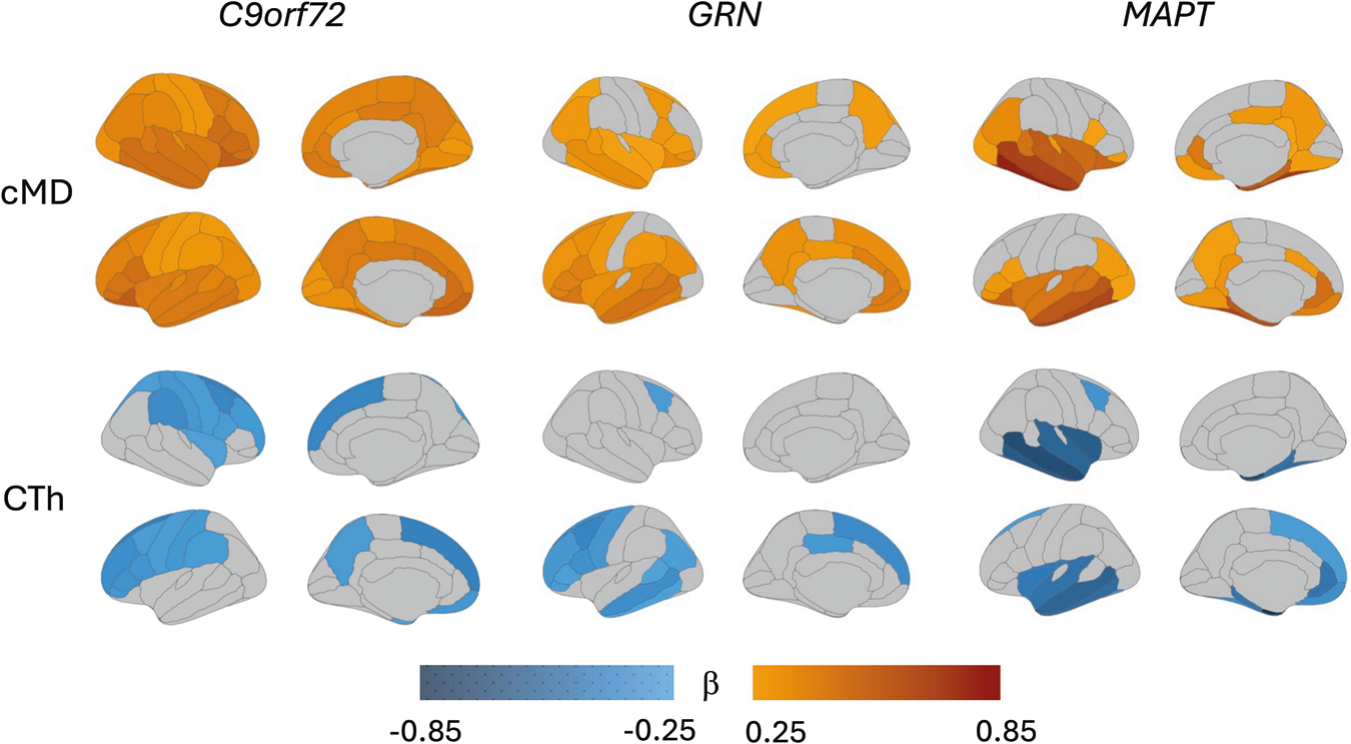
Brain maps illustrating the interaction of mutation status (carrier vs non-carrier) with age on the regional cMD and CTh as dependent variables. The top panel displays β coefficients from linear mixed-effects models indicating regions with a significant interaction effect, meaning that there was a significantly different trajectory of cMD vs age in mutation carriers compared with that in non-carriers (β coefficients depicted with red tones). The lower panel displays β coefficients from linear mixed-effects models for the respective interaction effect for CTh (blue tones). The scale represents β values ranging from 0.25 (light orange) to 0.85 (dark red) for cMD, and from −0.25 (light blue) to −0.85 (dark blue) for CTh. All analyses in mutation carriers are presented stratified by mutation type (*C9orf72*, *GRN* and *MAPT*). Only regions with *P*-values adjusted for multiple comparisons <0.05 (two-sided tests) are coloured. cMD cortical mean diffusivity; CTh cortical thickness.

The multimodal neuroimaging analysis revealed substantial differences in regional cMD and CTh findings by mutation type (Fig. 1). In the whole study sample (*n* = 710), the interaction analysis demonstrated that the association of each neuroimaging modality, cMD or CTh, with age was significantly stronger in mutation carriers than in non-carriers for all mutation types, but with different topographical patterns; the coloured regions on the brain maps represent significant results after correction for multiple comparisons (Fig. 1). Brain regions with significant interaction term had higher effect size (β coefficients, Fig. 1) and were more widespread for cMD than for CTh.

In *C9orf72* carriers, cMD in all 68 ROIs showed a significant interaction effect, meaning that the association between regional cMD and age was significantly stronger in carriers than in non-carriers across the brain, with greatest effect size (β) in superior-frontal, extending over lateral orbitofrontal and middle-temporal regions. In contrast, for CTh only 56% of ROIs showed a significant interaction, with a peak effect localized at superior-frontal and caudal middle-frontal regions (Fig. 1). The proportion of brain regions with significant interaction effect was statistically greater for cMD than for CTh (χ^2^ = 38.5, *P* < 0.0001), meaning that cMD findings were statistically verified to be occupying a larger spatial extension over the cortex than respective CTh findings.

In *GRN* carriers, 96% of brain regions had a significant interaction for cMD, with peak β observed in the left lateral middle-temporal and bilateral orbitofrontal regions, compared with significant findings occupying 26% of ROIs for CTh (which peaked at left lateral middle-temporal and left superior- and middle-frontal regions); the topographical extension of significant cMD findings was statistically greater than that of CTh (χ^2^ = 68.2, *P* < 0.0001).

In *MAPT* carriers, 71% of ROIs had a significant interaction for cMD (with peak β observed in bilateral middle- and inferior-temporal regions, bilateral insular and entorhinal cortices, and bilateral inferior-parietal and precuneus). In comparison, a significant interaction was observed only for 29% of ROIs for CTh, which was more restricted to bilateral middle- and inferior-temporal lobe and insula; the difference between the topographical extensions of cMD and CTh findings was statistically significant (χ^2^ = 23.1, *P* < 0.0001).

### Cortical microstructure and macrostructure in presymptomatic and symptomatic carriers (Aim 2)

The imaging data revealed substantial differences in the topography of cMD and CTh by mutation type both for pMC and sMC, respectively vs non-carriers (Supplementary Fig. 1). In the analysis of the whole study sample (*n* = 710), 90% of the brain regions had elevated cMD (peaking at superior-parietal, insula, pars opercularis and precuneus) and 66% had reduced CTh (more focally observed at precuneus, and supramarginal regions) at presymptomatic stage for the *C9orf72* mutation type; the proportion of significant brain regions was significantly different for cMD compared with CTh (χ^2^ = 10.9, *P* = 0.0009). The *MAPT* pMC group had elevated cMD locally restricted to the entorhinal cortex, with no alterations in CTh observed in any ROI. The *GRN* pMC group had no cMD or CTh alterations in any ROI. Regional cMD was elevated in sMC across virtually the whole brain vs non-carriers for all mutation types, while cortical thinning involved significantly lower proportion of regions compared with regions of elevated cMD (84% in *C9orf72* carriers [χ^2^ = 12.0, *P* = 0.0005], 75% in *GRN* carriers [χ^2^ = 19.4, *P* < 0.0001], and 51% in *MAPT* carriers [χ^2^ = 43.6, *P* < 0.0001]) (Supplementary Fig. 1). For completeness, Supplementary Fig. 2 illustrates the respective comparison between sMC vs pMC, where the spatial extent of significant findings was significantly larger for cMD than CTh in all mutation types.

### Cortical microstructure and macrostructure across stages of disease severity (Aim 3)

**Fig. 2:**
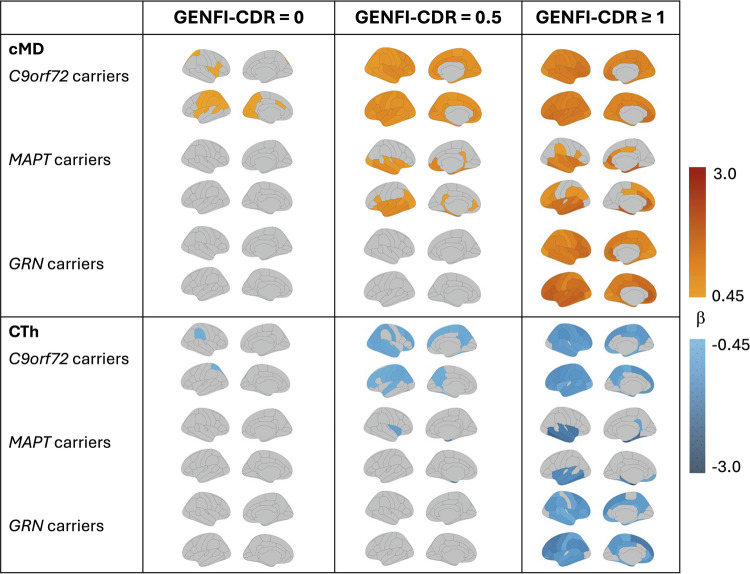
Brain maps illustrating the regional cMD and CTh topographical patterns in mutation carriers across disease stages defined by GENFI-CDR = 0, 0.5 and ≥ 1 vs non-carriers. The top panel displays β coefficients from linear mixed-effects models indicating increased cMD (red tones) in mutation carriers vs non-carriers, and the lower panel displays β coefficients from linear mixed-effects models indicating regions of reduced CTh (blue tones). The scale represents β values ranging from 0.45 (light orange) to 3.0 (dark red) corresponding to elevated cMD in mutation carriers vs non-carriers, and from −0.45 (light blue) to −3.0 (dark blue) indicating reduced CTh in mutation carriers vs non-carriers. All analyses in mutation carriers are presented stratified by mutation type (*C9orf72*, *GRN* and *MAPT*). Only regions with *P*-values adjusted for multiple comparisons < 0.05 (two-sided tests) are coloured. cMD cortical mean diffusivity; CTh cortical thickness; GENFI-CDR GENetic Frontotemporal dementia Initiative Clinical Dementia Rating scores.

To investigate more nuanced alterations in cMD and CTh across stages of disease severity we performed analyses with mutation carriers divided into stages of disease severity (GENFI-CDR = 0, 0.5 and ≥1), compared with non-carriers (Table 1, Fig. 2).

At GENFI-CDR = 0 stage, only *C9orf72* (and not *GRN* or *MAPT*) carriers showed elevated cMD involving 76% of the cortex including superior-parietal, insula and precuneus; in comparison, only 22% of the cortex was affected by thinning, mostly restricted to superior-parietal and supramarginal areas (χ^2^ = 40.3, *P* < 0.0001).

At GENFI-CDR = 0.5 stage, elevated cMD was observed in *C9orf72* carriers involving virtually the whole cortex as well as in *MAPT* carriers (affecting 44% of the cortex), while cortical thinning affected only 47% of ROIs in *C9orf72* carriers (significantly smaller extension than that of elevated cMD, χ^2^ = 49.0, *P* < 0.0001), and cortical thinning was only observed in the insular and entorhinal cortices in *MAPT* carriers, involving 7% of the cortex, which was significantly smaller than the extension of elevated cMD (χ^2^ = 24.0, *P* < 0.0001).

At GENFI-CDR ≥ 1 stage, mutation carriers of all types showed elevated regional cMD vs non-carriers, more pronounced (greater β) and widespread compared with respective areas of cortical thinning. For both cMD and CTh neuroimaging measures, *C9orf72* carriers had neuroimaging findings that were symmetric and extending over large parts of the frontal, temporal and parietal lobes. *GRN* carriers had asymmetric left-dominant pattern with strongest effect on left frontotemporal areas. *MAPT* carriers showed bilateral middle- and inferior-temporal cortical thinning, and elevated cMD in the same regions but also extending over larger areas including bilateral frontal, precuneus and anterior cingulate. Among the different mutation types, the spatial extent of elevated cMD was largest for the *C9orf72* and *GRN* mutation carrier types involving virtually the whole cortex, while respective cortical thinning affected significantly fewer areas (81%) for *C9orf72* (χ^2^ = 14.4, *P* = 0.0002) and 78% for *GRN* carriers (χ^2^ = 16.9, *P* < 0.0001) (Fig. 2). In *MAPT* carriers, elevated cMD extended over 57% of ROIs compared with only 26% of ROIs with cortical thinning, which was significantly smaller (χ^2^ = 13.3, *P* = 0.0003).

Taken together, these results on neuroimaging data across disease stages suggest that cMD is more sensitive than CTh to track early signs of cortical injury prior to overt loss of grey matter, with greatest sensitivity in *C9orf72* mutation carriers (starting at GENFI-CDR = 0), followed by *MAPT* carriers (starting at GENFI-CDR = 0.5), and only from late stages (GENFI-CDR ≥ 1) in *GRN* carriers. At all stages, the areas of significant cortical microstructural injury were more spatially extended than those affected by cortical thinning.

### Association of baseline cortical microstructure and macrostructure with longitudinal clinical progression (Aim 4)

**Fig. 3:**
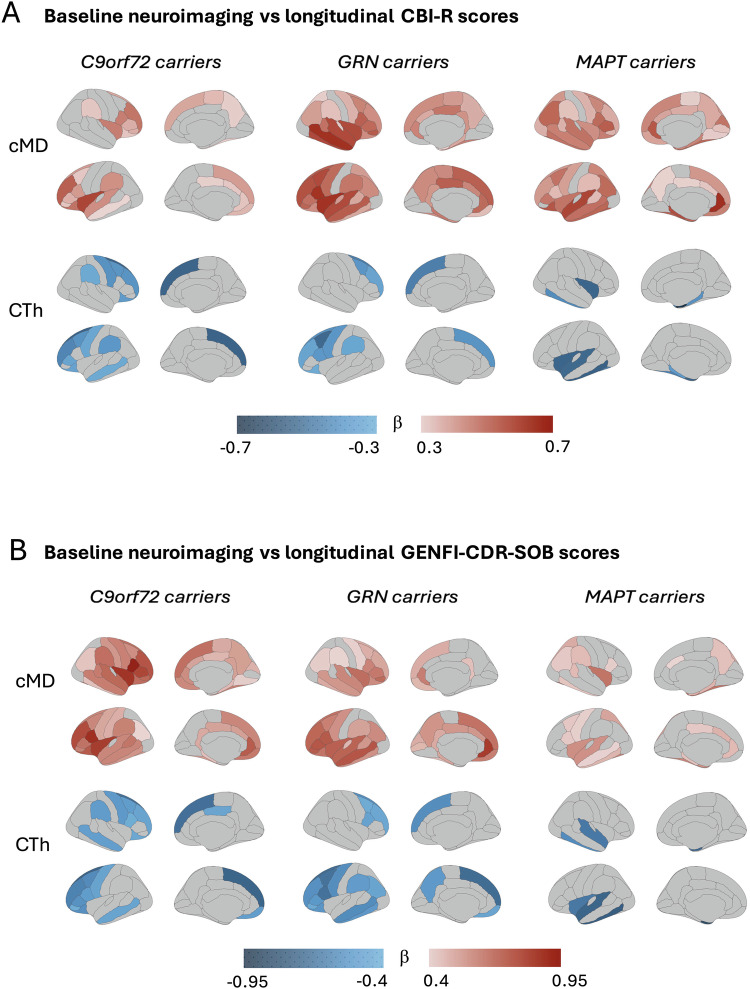
Brain maps depicting associations of regional cMD and CTh in mutation carriers at baseline with longitudinal clinical outcome measures. **A** Cambridge Behavioural Inventory-Revised (CBI-R), (**B**) CDR® plus NACC FTLD-NM SOB (GENFI-CDR-SOB). The brain maps display β coefficients from linear mixed-effects models indicating positive (red tones) or negative (blue tones) associations between baseline neuroimaging and longitudinal clinical data. For the associations involving baseline cMD, the scale represents β values ranging from 0.3 (light pink) to 0.7 (dark red) for CBI-R, and from 0.4 (light pink) to 0.95 (dark red) for GENFI-CDR-SOB. For the associations involving baseline CTh and longitudinal clinical data, the scale represents β values ranging from −0.3 (light blue) to −0.7 (dark blue) for CBI-R, and from −0.4 (light blue) to −0.95 (dark blue) for GENFI-CDR-SOB. All analyses are presented stratified by mutation type (*C9orf72*, *GRN* and *MAPT*). Only regions with *P*-values adjusted for multiple comparisons < 0.05 (two-sided tests) are coloured. CBI-R Cambridge Behavioural Inventory-Revised; cMD cortical mean diffusivity; CTh cortical thickness; GENFI-CDR-SOB GENetic Frontotemporal dementia Initiative Clinical Dementia Rating Sum-of-Boxes.

**Fig. 4:**
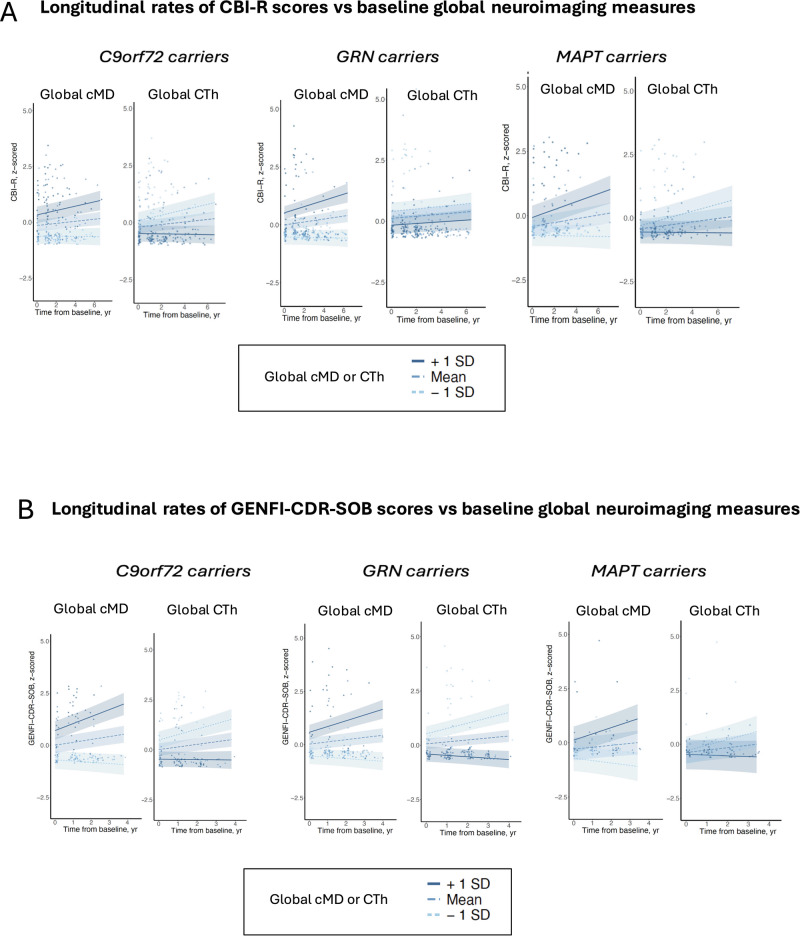
Interaction plots illustrating the moderating effect of global cMD and of global CTh at baseline in mutation carriers on the longitudinal evolution of clinical outcome measures. **A** Cambridge Behavioural Inventory-Revised (CBI-R), (**B**) CDR® plus NACC FTLD-NM SOB (GENFI-CDR-SOB). The regression lines represent model-predicted longitudinal clinical progression including 95% confidence bands, at three discrete levels of global cMD and of global CTh: mean, mean - 1 SD, and mean + 1 SD. All analyses are presented stratified by mutation type (*C9orf72*, *GRN* and *MAPT*). Individual dots represent repeated clinical visits. CBI-R Cambridge Behavioural Inventory-Revised; cMD cortical mean diffusivity; CTh cortical thickness; GENFI-CDR-SOB GENetic Frontotemporal dementia Initiative Clinical Dementia Rating Sum-of-Boxes; SD standard deviation.

In carriers stratified by mutation type, cMD_ROI_ at baseline was more strongly associated (higher absolute β coefficients) than CTh_ROI_ with longitudinal clinical outcome data, both for CBI-R (Fig. 3A) and GENFI-CDR-SOB (Fig. 3B). Statistical LMEM results for global baseline cMD and CTh predicting longitudinal clinical progression are presented on Table 2. The interaction terms of global cMD × time, and global CTh × time used in models predicting longitudinal clinical outcome data are illustrated in Fig. 4, with time measured in years from baseline to each longitudinal visit. While global cMD and CTh were entered as continuous predictors in the models (Eqs. 3), their moderating effect on the slope of clinical progression is illustrated for three discrete levels (mean, mean±1 SD) of baseline global neuroimaging measures for visualization purposes (Fig. 4).

**Table 2:** Statistical results of linear mixed-effects models predicting longitudinal clinical data.

LMEMs predicting longitudinal CBI-R scores (Eqs. 3)									
Models with global cMD as predictor	β (95% CI)	df	t	*P*	Models with global CTh as predictor	β (95% CI)	df	t	*P*
***C9orf72*** **mutation carriers**									
Global cMD	0.56 (0.31, 0.80)	68	4.5	<0.0001	Global CTh	−0.38 (−0.62, −0.14)	68	−3.1	0.0025
Time	0.08 (0.03, 0.12)	172	3.5	0.0007	Time	0.09 (0.05, 0.13)	172	4.1	0.0001
Global cMD x Time	0.08 (0.04, 0.13)	172	3.5	0.0007	Global CTh x Time	−0.10 (−0.15, −0.06)	172	−4.4	<0.0001
***GRN*** **mutation carriers**									
Global cMD	0.64 (0.46, 0.83)	82	6.8	<0.0001	Global CTh	−0.30 (−0.49, −0.10)	82	−2.9	0.0041
Time	0.11 (0.06, 0.16)	203	4.1	0.0001	Time	0.07 (0.02, 0.12)	203	2.8	0.0050
Global cMD x Time	0.12 (0.06, 0.18)	203	3.7	0.0003	Global CTh x Time	−0.02 (−0.07, 0.04)	203	−0.5	0.60
***MAPT*** **mutation carriers**									
Global cMD	0.47 (0.23, 0.72)	40	3.8	0.0006	Global CTh	−0.24 (−0.54, 0.05)	40	−1.6	0.11
Time	0.11 (0.07, 0.16)	113	4.8	<0.0001	Time	0.11 (0.06, 0.15)	113	4.4	<0.0001
Global cMD x Time	0.13 (0.07, 0.18)	113	4.7	<0.0001	Global CTh x Time	−0.11 (−0.17, −0.06)	113	−4.0	0.0001
**LMEMs predicting longitudinal GENFI-CDR-SOB scores (Eqs. 3)**									
**Models with global cMD as predictor**	**β (95% CI)**	**df**	**t**	***P***	**Models with global CTh as predictor**	**β (95% CI)**	**df**	**t**	***P***
***C9orf72*** **mutation carriers**									
Global cMD	0.88 (0.66, 1.11)	48	7.7	<0.0001	Global CTh	−0.61 (−0.86, −0.35)	48	−4.7	<0.0001
Time	0.13 (0.08, 0.18)	91	5.0	<0.0001	Time	0.12 (0.07, 0.18)	91	4.5	<0.0001
Global cMD x Time	0.19 (0.12, 0.25)	91	5.8	<0.0001	Global CTh x Time	−0.13 (−0.19, −0.07)	91	−4.4	<0.0001
***GRN*** **mutation carriers**									
Global cMD	0.76 (0.56, 0.96)	45	7.4	<0.0001	Global CTh	−0.66 (−0.90, −0.42)	45	−5.3	<0.0001
Time	0.11 (0.06, 0.17)	119	4.1	0.0001	Time	0.10 (0.05, 0.15)	119	3.6	0.0004
Global cMD x Time	0.18 (0.12, 0.25)	119	5.4	<0.0001	Global CTh x Time	−0.17 (−0.23, −0.11)	119	−5.4	<0.0001
***MAPT*** **mutation carriers**									
Global cMD	0.64 (0.33, 0.94)	23	4.1	0.0004	Global CTh	−0.25 (−0.68, 0.19)	23	−1.1	0.29
Time	0.09 (0.001, 0.18)	59	2.0	0.052	Time	0.11 (0.02, 0.21)	59	2.4	0.022
Global cMD x Time	0.20 (0.11, 0.30)	59	4.2	0.0001	Global CTh x Time	−0.15 (−0.25, −0.05)	59	−2.8	0.0068

Global cMD (cortical mean diffusivity) and global CTh (cortical thickness) represent the average of 68 regional cMD or 68 regional CTh values, respectively. The independent predictor “Time” corresponds to time from baseline to each of the longitudinal visits, in years. Global cMD × Time and Global CTh × Time are interaction terms used in the models. All models included age at baseline, sex and education as covariates, and individual nested within GENFI site as random intercept.

*β* standardized β (standardized fixed-effects coefficients in the linear mixed-effects models); *CBI-R* Cambridge Behavioural Inventory-Revised; *CDR* Clinical Dementia Rating (same as GENFI-CDR); *CI* confidence interval; *cMD* cortical mean diffusivity; *CTh* cortical thickness; *GENFI-CDR-SOB* GENFI Clinical Dementia Rating Sum-of-Boxes; *df* degrees of freedom; *LMEM* linear mixed-effects model.

In *C9orf72* carriers, higher cMD in bilateral frontotemporal cortex (insula, rostral middle-frontal, superior-frontal) as well as in bilateral parietal regions (precuneus) was associated with longitudinal increase in both CBI-R and GENFI-CDR-SOB scores, while more restricted frontotemporal regions of lower CTh were associated with subsequent clinical progression (Fig. 3). Global baseline cMD in *C9orf72* carriers had greater ability than CTh to predict subsequent clinical progression. Global baseline cMD had a stronger absolute effect as measured by β (95% CI, *P*-value)= 0.56 (0.31 to 0.80, *P* < 0.0001) to predict longitudinal CBI-R, compared with global baseline CTh that had a respective β (95% CI, *P*-value)= −0.38 (−0.62 to −0.14, *P* = 0.0025). Also, global baseline cMD had a β (95% CI, *P*-value)= 0.88 (0.66 to 1.11, *P* < 0.0001) to predict longitudinal GENFI-CDR-SOB, which was a stronger effect compared with global baseline CTh that had a β (95% CI, *P*-value)= −0.61 (−0.86 to −0.35, *P* < 0.0001). Both global cMD and CTh had a significant interaction with time in predicting steeper clinical progression in *C9orf72* carriers (Fig. 4).

In *GRN* carriers, substantially more widespread areas of higher cMD, primarily frontotemporal regions, were predictive of greater longitudinal scores in both CBI-R and GENFI-CDR-SOB, while significant regions of lower CTh were more localized to fewer frontal and lateral inferior-temporal areas (Fig. 3). Global baseline cMD had a β (95% CI, *P*-value)= 0.64 (0.46 to 0.83, *P* < 0.0001) to predict longitudinal CBI-R, compared with global baseline CTh that had a respective β (95% CI, *P*-value)= −0.30 (−0.49 to −0.10, *P* = 0.0041). Also, global baseline cMD had a β (95% CI, *P*-value)= 0.76 (0.56 to 0.96, *P* < 0.0001) to predict longitudinal GENFI-CDR-SOB, which was a stronger effect compared with global baseline CTh that had a respective β (95% CI, *P*-value)= −0.66 (−0.90 to −0.42, *P* < 0.0001) (Table 2). Only global cMD at baseline, and not global CTh, had a significant interaction with time and thus predicted steeper slope of CBI-R scores, while both global cMD and CTh were associated with steeper slope of GENFI-CDR-SOB (Table 2, Fig. 4).

In *MAPT* carriers, the regions of baseline cMD that significantly predicted longitudinal clinical progression were substantially more widespread than for CTh, which were restricted to fewer lateral-temporal cortical areas. When using global neuroimaging measures, only global baseline cMD (and not CTh) was associated with longitudinal clinical progression. Global baseline cMD had a β (95% CI, *P*-value)= 0.47 (0.23 to 0.72, *P* = 0.0006) to predict longitudinal CBI-R, and a β (95% CI, *P*-value)= 0.64 (0.33 to 0.94, *P* = 0.0004) to predict longitudinal GENFI-CDR-SOB (Table 2). Both global cMD and global CTh had a significant interaction with time and thus predicted steeper slopes of CBI-R and GENFI-CDR-SOB (Fig. 4).

Age at baseline, sex and education covariates did not have significant contributions to longitudinal clinical progression in any of the LMEMs in mutation carriers, except for education which had a protective effect in *GRN* carriers with respect to CBI-R longitudinal evolution. Of note, we repeated all longitudinal models within the non-carrier group, and no brain region of baseline cMD or CTh showed an association with longitudinal CBI-R or GENFI-CDR-SOB.

Also, we repeated the linear mixed-effects models (Eq. 3a) by including both global cMD × time and global CTh × time as predictors together in the same model, to compare their respective abilities to predict longitudinal CBI-R (Supplementary Table 3) and longitudinal GENFI-CDR-SOB (Supplementary Table 4). For all mutation carrier types, global cMD remained a significant predictor of longitudinal CBI-R scores even after including global CTh as independent predictor, and global CTh did not significantly explain longitudinal CBI-R. For longitudinal GENFI-CDR-SOB, global cMD remained a significant predictor for all three mutation carrier types, while global CTh was not a significant predictor for *C9orf72* or for *MAPT* carrier types. For *GRN* carriers, the β coefficient of global cMD (β = 0.59 [0.36, 0.82], *P* < 0.0001) was higher than that of global CTh (β = −0.31 [−0.56, −0.06], *P* = 0.018).

Finally, we performed a sensitivity analysis to check the ability of cMD and CTh to predict clinical progression over a shorter longitudinal clinical follow-up (average of 1.1 ± 0.1 years), which is common in clinical trials of FTD [30]. The demographic information of this subset is presented in Supplementary Table 5, and the statistical results are presented in Supplementary Table 6. Global cMD at baseline remained a significant predictor of longitudinal CBI-R scores, with greater β coefficient than global CTh for all mutation carrier types; of note, global CTh was not a significant predictor of longitudinal CBI-R in *MAPT* carriers. Global cMD at baseline remained a significant predictor of longitudinal GENFI-CDR-SOB scores for *C9orf72* and *GRN* mutation types, with greater β coefficient than global CTh. However, neither global cMD nor global CTh significantly predicted longitudinal GENFI-CDR-SOB scores in *MAPT* carriers, which may be in part due to sample size limitations in the *MAPT* carrier group in this sub-analysis (*n* = 29).

## Discussion

Novel non-invasive neuroimaging and fluid biomarkers are transforming FTD diagnosis and may become affordable and scalable pre-screening tools. In particular, cortical microstructure may be a more sensitive sign of brain injury than cortical thinning as previously shown in the Alzheimer continuum [15, 21], but data on cortical microstructural imaging in genetic forms of FTD are still lacking. In addition, whether cortical microstructure is associated with disease severity and subsequent clinical progression in genetic FTD is unknown. In this study, we analysed the brain regional patterns of cortical mean diffusivity (cMD) side-by-side with cortical thickness (CTh) in genetic FTD, stratified by mutation type.

Firstly, we found evidence for heterogeneity of cortical microstructural patterns across mutation types. Also, within each mutation type, there was a stronger interaction of mutation status (carrier vs non-carrier) × age on cMD_ROI_ than on CTh_ROI_, involving more widespread brain regions. Elevated cMD_ROI_ was observed from early stages of disease progression, with variation due to mutation type. The earliest elevations in cMD were observed already at GENFI-CDR = 0 in *C9orf72* carriers involving the insula, superior-parietal and precuneus, which became more widespread at subsequent stages. In *MAPT* carriers, the earliest cMD elevation was noted at GENFI-CDR = 0.5 involving insula and entorhinal areas, while elevated cMD was apparent only at GENFI-CDR ≥ 1 stage for *GRN* carriers affecting frontotemporal regions including the insula. In all mutation types, corresponding cortical thinning patterns were less spatially extended and had lower effect size.

Our study has found that the cMD and CTh topographical patterns differ across genetic mutation types, corroborating previous studies on volumetrics in genetic FTD [10, 31, 32], with cMD likely reflecting subtle neurodegeneration. We also observed regional commonalities, where cortical microstructural alterations affected the fronto-insular-anterior cingulate cortices in all mutation types, a region known to be part of the salience network and considered to be the epicenter of FTD [33], thus the observed cortical microstructural alterations might reflect injury to this network prior to overt atrophy.

Previous studies reported that the earliest biomarker alterations in *C9orf72* carriers are structural MRI changes followed by elevated plasma neurofilament light concentrations [10]. Our study adds evidence on very early cortical microstructural injury in *C9orf72* carriers, extending beyond areas affected by atrophy, suggesting that cortical microstructural injury is among the earliest biomarker for this mutation type, with added value as sensitive and prognostic biomarker beyond macrostructural MRI. Our findings in *GRN* carriers are consistent with previous reports where *GRN* structural changes begin only at around symptom onset, and rapidly progress thereafter [10, 31]. In our study, the lack of observed presymptomatic grey matter changes in *GRN* carriers may be partly attributed to heterogeneous asymmetric structural alterations, with subgroups of individuals being left- or right-dominant, and our findings are consistent with previous reports [34]. In *GRN* carriers, we observed greater microstructural alterations on the left side and a stronger association of left cortical microstructural alterations with longitudinal cognitive decline; these observations provide first data using cortical microstructure, consistent with previous reports of greater proportion of *GRN* carriers with left-dominant over right-dominant neurodegenerative changes, where left-dominant *GRN* carriers also have a faster rate of disease progression [34]. Our findings suggest that cortical microstructure may be a valuable tool to aid in the stratification of individuals for clinical trials of *GRN* carriers [30]

Early sensitive biomarkers of cortical injury are still lacking in the field of FTD and are urgently needed for differential diagnosis and prognosis, and for selection of at-risk individuals for preventive trials [10, 35]. Our findings support the value of cMD as more sensitive biomarker of cortical injury than CTh, which may be capturing early synaptic injury prior to measurable thinning. We also showed that regional alterations in cortical microstructure are associated with longitudinal clinical outcome measures and thus demonstrated that the microstructural alterations may have an impact on subsequent disease progression, even in the absence of overt atrophy. In our study, global baseline cMD was more strongly predictive than CTh of subsequent clinical progression in all mutation types, noting that for the *MAPT* carriers only global baseline cMD (and not CTh) predicted subsequent longitudinal clinical outcome scores. The robustness of the predictive ability of cortical microstructure is further supported by the finding that covariates (age at baseline, sex and education) did not significantly contribute to explaining longitudinal clinical progression.

Future studies are warranted to further explore the clinical relevance of asymmetric cortical microstructural alterations and their prognostic implications, and the association of cortical microstructure with other neuroimaging and fluid biomarkers of neuronal injury (such as plasma neurofilament light [NfL]), gliosis (plasma glial fibrillary acidic protein [GFAP]), neuroinflammation, proteinopathy and synaptic damage [36–38], across different mutation types in genetic FTD, and in sporadic forms of FTD with diverse clinical phenotypes. Previous studies found that plasma NfL and GFAP markers are elevated in genetic FTD, with NfL outperforming GFAP in their ability to discriminate carriers from non-carriers, and in their ability to predict clinical progression [39]. In sporadic FTD (*n* = 32 patients), a positive association was reported between NfL in CSF and cMD in prefrontal, temporal and parietal brain regions [17]; this finding needs to be tested in genetic forms of FTD. Future studies investigating the associations between cMD, NfL and GFAP will allow to assess whether cortical microstructure is more closely associated with neuronal (NfL) or glial (GFAP) mechanisms.

The neuropathology of genetic FTD is highly heterogeneous involving several types of TDP-43 and tau deposits [3, 40, 41], which are all potentially associated with the observed cortical microstructural alterations. Other pathological alterations such as synaptic loss, gliosis or microvacuolar changes have been reported in cortical regions in genetic FTD [3], that may also contribute to explaining the cortical microstructural findings. Multimodal microstructural and PET imaging studies using novel specific PET tracers for the different types of proteinopathy, gliosis or synaptic density in genetic FTD will be needed, as well as correlative studies between in vivo microstructural imaging and postmortem histopathology. It will also be interesting in the future to compare cortical microstructural imaging with other potentially sensitive neuroimaging measures of cortical complexity such as the quantification of the gyrification index, sulcal depth and fractal dimension, as previous studies have suggested an early reduction in cortical complexity in neurodegenerative diseases [42].

Key strengths of our study include the investigation of a novel cortical microstructural imaging approach in the largest sample of genetic FTD mutation carriers and non-carriers currently available, that extends from presymptomatic to symptomatic stages, and the investigation at different stages of disease severity as measured by the recently validated GENFI-CDR clinical measure [23], which is specific to FTD, including behavioural, language, neuropsychiatric and motor domains. Also, our study compares cortical microstructure and thickness side-by-side in the same individuals, which allows for a direct comparison of their respective topographical alterations, and their associations with longitudinal clinical progression. The longitudinal follow-up time during an average of 2-3 years is a typical time frame for clinical trials, pointing to potential utility of cortical microstructure to select participants in clinical trials. We replicated our analyses for a shorter follow-up time of about one year, suggesting that cortical microstructure has predictive ability also in shorter trials. The multicentre GENFI protocol assures that scan protocols are harmonized across centres, which provides a consistent and robust database [10]. The use of only 3-Tesla images, extensive data quality control after each preprocessing step and careful exclusion of outliers support the robustness of the findings. Due to differences in age between pMC vs NC and between sMC vs NC, age was incorporated as covariate in all models. In this regard, the finding of increased cMD in pMC is notable given the younger age of this group compared with the non-carriers, supporting the concept that increased cMD is a marker of brain pathology due to the FTD mutations and not normal ageing.

Our study has some limitations. Regarding the investigation of the temporal order of microstructural and macrostructural alterations, with our cross-sectional neuroimaging data we could conclude that regional cMD alterations are observed at earlier stages of disease progression than respective CTh alterations, based on findings at group level and cross-sectionally only. To further investigate whether cMD temporally precedes CTh alterations in given ROIs in the same individuals, it would be necessary to analyze longitudinal structural and diffusion-weighted MRI data over multiple time points. While the cortical microstructural quantification in this study is designed to minimize partial volume effects by using techniques for accurate co-registration and sampling in the midpoint of the cortical ribbon, extracting cMD from the cortex is still prone to possible partial volume effects due to the limited resolution of diffusion-weighted MRI scans. Thus, part of the effects could be driven by partial inclusion of CSF in the diffusion-weighted MRI voxels and not by microstructural changes alone. While we substantially reduced the possibility of Type I error due to performing multiple models across 68 ROIs by using appropriate FDR methods, the complete removal of false positives is not absolutely guaranteed. However, a strength of our study was that by comparing the statistical models for cMD and CTh neuroimaging variables side-by-side, we found greater effects (β coefficients and 95% CIs) and greater topographical extensions for cMD compared with CTh within the same group of individuals, both for cross-sectional group comparisons and in the prediction of longitudinal clinical progression. The *MAPT* group had a smaller sample size and fewer longitudinal follow-up visits compared with the *C9orf72* and the *GRN* groups, which could have somewhat limited the statistical power of the findings in *MAPT* carriers. This limitation could be especially relevant for the associations between neuroimaging and clinical follow-up data, as the clinical outcome data have a large inherent variability that requires larger sample sizes. Despite these sample size differences, cortical microstructure was a prognostic marker of longitudinal clinical progression across all three mutation types, supporting the robustness of the findings. While our findings provide strong support for cMD as a biomarker of disease stage and prognosis of clinical outcomes, to use DTI neuroimaging tools in clinical practice and in therapeutic trials more research is needed on the translation of our group-based results to the individual patient level and further including longitudinal neuroimaging observations.

This study provides evidence of global, regional and gene-specific cMD changes from early presymptomatic stages in genetic FTD, making cMD a promising sensitive neuroimaging biomarker of disease progression and for selection of individuals at risk of short-term decline for enrollment in therapeutic trials.

## Supplementary information

Supplementary information

## Data Availability

Data will be shared according to the GENFI data sharing agreement, after review by the GENFI data access committee with final approval granted by the GENFI steering committee. A code developed in-house was used for the processing and quantification of cortical microstructure from diffusion-weighted MRI images, and the code (Diffusion ON SURFace, Victor Montal) is openly available in GitHub: https://gitlab.com/vmontalb/diffusion-on-surface.
