# Supplementary material for: Cortical microstructure is associated with disease severity and clinical progression in genetic frontotemporal dementia: a GENFI study
Source: Mol Psychiatry. 2025 Oct 9;30(12):5800–12. doi: 10.1038/s41380-025-03280-x (PMC12602311; doi:10.1038/s41380-025-03280-x)
Supplement: Supplementary file 1 — Supplementary information [file 41380_2025_3280_MOESM1_ESM.pdf]

Supplementary information

Cortical microstructure is associated with disease severity and clinical progression in genetic frontotemporal dementia: a GENFI study

Elena Rodriguez-Vieitez, Melissa T. Rydell, Abbe Ullgren, Victor Montal, Ignacio Illán-Gala, Juan Fortea, Vesna Jelic, Arabella Bouzigues, Lucy L. Russell, Phoebe H. Foster, Eve Ferry-Bolder, John C. van Swieten, Lize C. Jiskoot, Harro Seelaar, Raquel Sanchez-Valle, Robert Laforce, Daniela Galimberti, Rik Vandenberghe, Alexandre de Mendonça, Pietro Tiraboschi, Isabel Santana, Alexander Gerhard, Johannes Levin, Sandro Sorbi, Markus Otto, Florence Pasquier, Simon Ducharme, Chris R. Butler, Isabelle Le Ber, Elizabeth Finger, Maria Carmela Tartaglia, Mario Masellis, James B. Rowe, Matthis Synofzik, Fermin Moreno, Barbara Borroni, Jonathan D. Rohrer, Eric Westman, Caroline Graff, on behalf of the Genetic Frontotemporal Dementia Initiative (GENFI)

Contents

Supplementary methods

Supplementary references

Supplementary tables

Supplementary Table 1 Demographic information of the whole study sample (*n*=710)

Supplementary Table 2 Demographic information of the subsets with longitudinal clinical data

Supplementary Table 3 Linear mixed-effects models of global cMD × time at baseline predicting longitudinal CBI-R data, including global CTh × time at baseline as independent predictor

Supplementary Table 4 Linear mixed-effects models of global cMD × time at baseline predicting longitudinal GENFI-CDR-SOB data, including global CTh × time at baseline as independent predictor

Supplementary Table 5 Demographic information for the subset of individuals with an average longitudinal clinical follow-up time of 1.1 (0.1) years

Supplementary Table 6 Statistical results of linear mixed-effects models predicting longitudinal clinical data in mutation carriers with an average longitudinal clinical follow-up time of 1.1 (0.1) years

Supplementary figures

Supplementary Fig. 1 Brain maps illustrating the regional cMD and CTh topographical patterns in mutation carriers (presymptomatic [pMC] and symptomatic [sMC] mutation carriers) vs non-carriers as controls

Supplementary Fig. 2 Brain maps illustrating the regional cMD and CTh topographical patterns in symptomatic (sMC) vs presymptomatic (pMC) mutation carriers

List of GENFI consortium co-investigators

Supplementary methods

Study participants and clinical assessment

The GENetic Frontotemporal dementia Initiative (GENFI) is a longitudinal multicentre cohort study of genetic FTD across Europe and Canada, investigating carriers of the *C9orf72*, *GRN* or *MAPT* mutations and their healthy first-degree relatives, following a standardized protocol. The inclusion criteria in GENFI are being an adult (>18 years of age) and a first-degree relative to a patient with genetic FTD or having been diagnosed with genetic FTD according to clinical criteria [1, 2]. For the present study, data were included from the fifth GENFI data freeze, in which participants from confirmed genetic FTD families were recruited between January 2012 and May 2019. Clinical status was determined according to established diagnostic criteria, based on this assessment and information from a structured interview with knowledgeable informants, including questions about behavioural, neuropsychiatric, cognitive, instrumental activities of daily living, motor, and autonomic symptoms as previously described [3, 4]. Both researchers and participants were blinded to the results of the genetic testing. Based on this clinical assessment, mutation carriers are classified as either presymptomatic (pMC) or symptomatic (sMC). First-degree relatives to an FTD patient without a disease-causing mutation are non-carriers (NC).

Supplementary references

1. Rascovsky K, Hodges JR, Knopman D, Mendez MF, Kramer JH, Neuhaus J, et al. Sensitivity of revised diagnostic criteria for the behavioural variant of frontotemporal dementia. Brain. 2011;134:2456–2477.

2. Gorno-Tempini ML, Hillis AE, Weintraub S, Kertesz A, Mendez M, Cappa SF, et al. Classification of primary progressive aphasia and its variants. Neurology. 2011;76:1006–1014.

3. Meeter LH, Kaat LD, Rohrer JD, Van Swieten JC. Imaging and fluid biomarkers in frontotemporal dementia. Nat Rev Neurol. 2017;13:406–419.

4. Rohrer JD, Nicholas JM, Cash DM, Van Swieten J, Dopper E, Jiskoot L, et al. Presymptomatic cognitive and neuroanatomical changes in genetic frontotemporal dementia in the Genetic Frontotemporal dementia Initiative (GENFI) study: a cross-sectional analysis. The Lancet Neurology. 2015;14:253–262.

Supplementary Table 1 Demographic information of the whole study sample (*n*=710)

	Non-carriers	*C9orf72* carriers			*GRN* carriers			*MAPT* carriers		
	NC	pMC	sMC	*P*-values	pMC	sMC	*P*-values	pMC	sMC	*P*-values
No. of participants, *n*	287	121	60		129	33		55	25	
Age, mean yr (SD)	46.4 (13.5)	43.3 (11.3)	62.8 (7.2)	pMC vs NC: *P*=0.02<br>sMC vs NC: *P*<0.001	45.9 (12.3)	62.4 (7.8)	sMC vs NC: *P*<0.001	40.4 (11.0)	57.2 (8.6)	pMC vs NC: *P*<0.001<br>sMC vs NC: *P*<0.001
Sex, female *n* (% female)	167 (58.2%)	72 (59.5%)	24 (40.0%)	sMC vs NC: *P*=0.015	81 (62.8%)	17 (51.5%)		31 (56.3%)	10 (40.0%)	
Education, mean yr (SD)	14.3 (3.3)	14.3 (3.1)	12.9 (3.5)	sMC vs NC: *P*=0.004	14.6 (3.4)	12.3 (3.2)	sMC vs NC: *P*=0.002	14.2 (3.1)	13.6 (3.7)	

The table includes statistical comparisons of demographic data for the comparisons between the presymptomatic mutation carriers (pMC) vs non-carriers (NC) and between symptomatic mutation carriers (sMC) vs non-carriers (NC) groups, stratified by gene type *C9orf72*, *GRN* and *MAPT*. *P*-values correspond to pairwise t-tests for continuous variables and χ^2-tests for categorical variables. No.= number; pMC = presymptomatic mutation carriers; SD = standard deviation; sMC = symptomatic mutation carriers; yr = year

Supplementary Table 2 Demographic information of the subsets with longitudinal clinical data

Subset (*n*=403) with longitudinal CBI-R data

Properties	Non-carriers	*C9orf72* carriers	*GRN* carriers	*MAPT* carriers
No. of participants, *n*	164	89	97	53
Age, mean yr (SD)	47.8 (13.3)	50.2 (13.1)	49.8 (13.2)	45.0 (13.7)
Sex, female *n* (% female)	101 (61.6%)	48 (53.9%)	62 (63.9%)	27 (50.9%)
Education, mean yr (SD)	14.7 (3.4)	14.0 (3.2)	14.5 (3.9)	14.3 (3.5)
pMC / sMC at baseline	-	60 / 29	80 / 17	38 / 15
CBI-R at baseline, mean (SD)	4.1 (6.2)	25.9 (33.3)	10.9 (20.9)	19.9 (32.1)
Total CBI-R follow-up time, mean yr (SD)	2.9 (1.7)	2.6 (1.6)	2.9 (1.5)	2.8 (1.4)

Subset (*n*=261) with longitudinal GENFI-CDR-SOB data

Properties	Non-carriers	*C9orf72* carriers	*GRN* carriers	*MAPT* carriers
No. of participants, *n*	97	69	60	35
Age, mean yr (SD)	44.7 (13.0)	49.6 (14.3)	48.0 (13.9)	42.3 (13.5)
Sex, female *n* (% female)	58 (59.8%)	39 (56.5%)	35 (58.3%)	19 (54.3%)
Education, mean yr (SD)	14.9 (3.5)	13.8 (3.4)	15.1 (4.1)	14.7 (2.6)
pMC / sMC at baseline	-	45 / 24	47 / 13	29 / 6
GENFI-CDR at baseline, mean (SD)	0.1 (0.2)	0.8 (1.0)	0.4 (0.7)	0.3 (0.5)
GENFI-CDR-SOB at baseline, mean (SD)	0.3 (0.7)	3.9 (6.0)	1.9 (4.2)	1.2 (2.6)
Total GENFI-CDR-SOB follow-up time, mean yr (SD)	2.0 (0.8)	1.7 (0.8)	2.3 (0.9)	2.1 (0.7)

CBI-R = Cambridge Behavioural Inventory-Revised; CDR = Clinical Dementia Rating (same as GENFI-CDR); GENFI-CDR-SOB = GENFI Clinical Dementia Rating Sum-of-Boxes; No. = number; pMC = presymptomatic mutation carrier; SD = standard deviation; sMC = symptomatic mutation carrier

Supplementary Table 3 Linear mixed-effects models of global cMD × time at baseline predicting longitudinal CBI-R data, including global CTh × time at baseline as independent predictor

Independent predictors	β (95% CI)	df	t	*P*
***C9orf72* mutation carriers**				
Global cMD	0.49 (0.19, 0.80)	67	3.2	0.0024
Global CTh	-0.11 (-0.39, 0.17)	67	-0.8	0.44
Time	0.09 (0.05, 0.13)	171	4.0	0.0001
Global cMD x Time	0.02 (-0.04, 0.08)	171	0.6	0.54
Global CTh x Time	-0.09 (-0.15, -0.03)	171	-2.8	0.0052
***GRN* mutation carriers**				
Global cMD	0.63 (0.41, 0.84)	81	5.7	<0.0001
Global CTh	-0.03 (-0.24, 0.17)	81	-0.3	0.75
Time	0.11 (0.06, 0.16)	202	4.2	<0.0001
Global cMD x Time	0.14 (0.07, 0.21)	202	3.8	0.0002
Global CTh x Time	0.03 (-0.03, 0.10)	202	1.1	0.28
***MAPT* mutation carriers**				
Global cMD	0.45 (0.18, 0.71)	39	3.3	0.0021
Global CTh	-0.07 (-0.36, 0.22)	39	-0.5	0.64
Time	0.11 (0.07, 0.16)	112	4.7	<0.0001
Global cMD x Time	0.10 (0.02, 0.17)	112	2.6	0.011
Global CTh x Time	-0.04 (-0.12, 0.04)	112	-1.1	0.29

Global cMD (cortical mean diffusivity) and global CTh (cortical thickness) represent the average of 68 regional cMD or 68 regional CTh values, respectively. The independent predictor "Time" corresponds to time from baseline to each of the longitudinal visits, in years. Global cMD × Time and Global CTh × Time are interaction terms used in the models. All models included age at baseline, sex and education as covariates, and individual nested within GENFI site as random intercept. β = standardized β (standardized fixed-effects coefficients in the linear mixed-effects models); CBI-R = Cambridge Behavioural Inventory-Revised; CI = confidence interval; cMD = cortical mean diffusivity; CTh = cortical thickness; df = degrees of freedom

Supplementary Table 4 Linear mixed-effects models of global cMD × time at baseline predicting longitudinal GENFI-CDR-SOB data, including global CTh × time at baseline as independent predictor

Independent predictors	β (95% CI)	df	t	*P*
		***C9orf72* mutation carriers**		
Global cMD	0.89 (0.54, 1.24)	47	4.9	<0.0001
Global CTh	0.01 (-0.32, 0.34)	47	0.1	0.96
Time	0.13 (0.08, 0.18)	90	5.0	<0.0001
Global cMD x Time	0.19 (0.08, 0.29)	90	3.3	0.0012
Global CTh x Time	-0.002 (-0.10, 0.10)	90	-0.05	0.96
		***GRN* mutation carriers**		
Global cMD	0.59 (0.36, 0.82)	44	5.0	<0.0001
Global CTh	-0.31 (-0.56, -0.06)	44	-2.5	0.018
Time	0.11 (0.06, 0.17)	118	4.2	0.0001
Global cMD x Time	0.11 (0.03, 0.20)	118	2.6	0.010
Global CTh x Time	-0.11 (-0.19, -0.03)	118	-2.7	0.0078
		***MAPT* mutation carriers**		
Global cMD	0.62 (0.31, 0.93)	22	3.9	0.0008
Global CTh	-0.09 (-0.49, 0.31)	22	-0.5	0.65
Time	0.10 (0.01, 0.18)	58	2.1	0.039
Global cMD x Time	0.18 (0.07, 0.28)	58	3.2	0.0024
Global CTh x Time	-0.06 (-0.17, 0.05)	58	-1.1	0.27

Global cMD (cortical mean diffusivity) and global CTh (cortical thickness) represent the average of 68 regional cMD or 68 regional CTh values, respectively. The independent predictor "Time" corresponds to time from baseline to each of the longitudinal visits, in years. Global cMD × Time and Global CTh × Time are interaction terms used in the models. All models included age at baseline, sex and education as covariates, and individual nested within GENFI site as random intercept. β = standardized β (standardized fixed-effects coefficients in the linear mixed-effects models); CI = confidence interval; cMD = cortical mean diffusivity; CTh = cortical thickness; df = degrees of freedom; GENFI-CDR-SOB = GENFI Clinical Dementia Rating Sum-of-Boxes.

Supplementary Table 5 Demographic information for the subset of individuals with an average longitudinal clinical follow-up time of 1.1 (0.1) years

Subset (*n*=279) with longitudinal CBI-R data with an average follow-up time of 1.1 (0.1) years

**Properties**	**Non-carriers**	***C9orf72* carriers**	***GRN* carriers**	***MAPT* carriers**
No. of participants, *n*	112	68	61	38
Age, mean yr (SD)	46.3 (13.4)	51.8 (13.0)	50.1 (13.9)	44.7 (14.2)
Sex, female *n* (% female)	66 (58.9%)	32 (47.1%)	36 (59.0%)	21 (55.3%)
Education, mean yr (SD)	14.9 (3.6)	13.9 (3.4)	14.5 (4.6)	15.0 (3.0)
pMC / sMC at baseline	-	42 / 26	44 / 17	27 / 11
CBI-R at baseline, mean (SD)	4.9 (6.6)	29.7 (33.8)	16.0 (25.0)	18.1 (31.0)
Total CBI-R follow-up time, mean yr (SD)	1.1 (0.1)	1.1 (0.1)	1.1 (0.1)	1.1 (0.1)

Subset (*n*=229) with longitudinal GENFI-CDR-SOB data with an average follow-up time of 1.1 (0.1) years

**Properties**	**Non-carriers**	***C9orf72* carriers**	***GRN* carriers**	***MAPT* carriers**
No. of participants, *n*	90	57	53	29
Age, mean yr (SD)	45.3 (12.6)	50.9 (13.9)	49.0 (14.1)	40.8 (13.2)
Sex, female *n* (% female)	54 (60.0%)	30 (52.6%)	31 (58.5%)	15 (51.7%)
Education, mean yr (SD)	14.9 (3.6)	14.0 (3.5)	15.1 (4.1)	15.2 (2.4)
pMC / sMC at baseline	-	35 / 22	40 / 13	24 / 5
GENFI-CDR at baseline, mean (SD)	0.1 (0.3)	0.9 (1.0)	0.5 (0.8)	0.3 (0.4)
GENFI-CDR-SOB at baseline, mean (SD)	0.3 (0.7)	4.3 (6.1)	2.1 (4.4)	1.2 (2.4)
Total GENFI-CDR-SOB follow-up time, mean yr (SD)	1.1 (0.1)	1.1 (0.2)	1.0 (0.1)	1.1 (0.1)

CBI-R = Cambridge Behavioural Inventory-Revised; CDR = Clinical Dementia Rating (same as GENFI-CDR); GENFI-CDR-SOB = GENFI Clinical Dementia Rating Sum-of-Boxes; No. = number; pMC = presymptomatic mutation carrier; SD = standard deviation; sMC = symptomatic mutation carrier

Supplementary Table 6 Statistical results of linear mixed-effects models predicting longitudinal clinical data in mutation carriers with an average longitudinal clinical follow-up time of 1.1 (0.1) years

LMEMs predicting longitudinal CBI-R scores (Eqs. 3)									
**Models with global cMD as predictor**	**β (95% CI)**	**df**	**t**	***P***	**Models with global CTh as predictor**	**β (95% CI)**	**df**	**t**	***P***
				***C9orf72* mutation carriers**					
Global cMD	0.54 (0.26, 0.82)	49	3.8	0.0004	Global CTh	-0.27 (-0.52, -0.02)	49	-2.1	0.043
Time	0.03 (-0.02, 0.07)	66	1.0	0.33	Time	0.02 (-0.03, 0.07)	66	2.0	0.34
Global cMD x Time	0.06 (0.01, 0.12)	66	2.5	0.017	Global CTh x Time	-0.05 (-0.1, -0.001)	66	-1.9	0.06
				***GRN* mutation carriers**					
Global cMD	0.64 (0.39, 0.88)	45	5.1	<0.0001	Global CTh	-0.44 (-0.70, -0.18)	45	-3.3	0.0019
Time	0.07 (0.02, 0.12)	59	2.7	0.0087	Time	0.07 (0.02, 0.13)	58	2.5	0.015
Global cMD x Time	0.09 (0.04, 0.14)	59	3.4	0.0014	Global CTh x Time	-0.04 (-0.10, 0.01)	58	-0.5	0.11
				***MAPT* mutation carriers**					
Global cMD	0.62 (0.31, 0.92)	26	4.0	0.0005	Global CTh	-0.26 (-0.65, 0.14)	26	-1.3	0.21
Time	0.03 (-0.009, 0.07)	36	1.5	0.15	Time	0.03 (-0.01, 0.06)	36	1.3	0.20
Global cMD x Time	0.04 (-0.004, 0.08)	36	1.8	0.084	Global CTh x Time	-0.03 (-0.07, 0.01)	36	-1.3	0.20

LMEMs predicting longitudinal GENFI-CDR-SOB scores (Eqs. 3)									
**Models with global cMD as predictor**	**β (95% CI)**	**df**	**t**	***P***	**Models with global CTh as predictor**	**β (95% CI)**	**df**	**t**	***P***
				***C9orf72* mutation carriers**					
Global cMD	0.89 (0.61, 1.16)	37	6.3	<0.0001	Global CTh	-0.51 (-0.79, -0.23)	37	-3.6	0.0009
Time	0.16 (0.10, 0.21)	55	5.4	<0.0001	Time	0.16 (0.09, 0.22)	54	4.9	<0.0001
Global cMD x Time	0.17 (0.11, 0.23)	55	5.4	<0.0001	Global CTh x Time	-0.12 (-0.19, -0.06)	54	-3.8	0.0004
				***GRN* mutation carriers**					
Global cMD	0.69 (0.50, 0.88)	38	7.0	<0.0001	Global CTh	-0.62 (-0.87, -0.37)	38	-4.8	<0.0001
Time	0.16 (0.10, 0.23)	51	4.9	0.0001	Time	0.16 (0.10, 0.22)	51	5.0	<0.0001
Global cMD x Time	0.17 (0.10, 0.23)	51	5.1	<0.0001	Global CTh x Time	-0.18 (-0.24, -0.12)	51	-5.7	<0.0001
				***MAPT* mutation carriers**					
Global cMD	-0.15 (-0.61, 0.31)	17	-0.6	0.54	Global CTh	-0.31 (-0.70, 0.08)	17	-1.5	0.14
Time	0.04 (-0.05, 0.14)	27	0.9	0.38	Time	0.01 (-0.07, 0.10)	26	0.3	0.77
Global cMD x Time	-0.02 (-0.12, 0.07)	27	-0.5	0.61	Global CTh x Time	-0.07 (-0.15, 0.01)	26	-1.6	0.11

Global cMD (cortical mean diffusivity) and global CTh (cortical thickness) represent the average of 68 regional cMD or 68 regional CTh values, respectively. The independent predictor "Time" corresponds to time from baseline to each of the longitudinal visits, in years. Global cMD × Time and Global CTh × Time are interaction terms used in the models. All models included age at baseline, sex and education as covariates, and individual nested within GENFI site as random intercept. β = standardized β (standardized fixed-effects coefficients in the linear mixed-effects models); CBI-R = Cambridge Behavioural Inventory-Revised; CDR = Clinical Dementia Rating (same as GENFI-CDR); CI = confidence interval; cMD = cortical mean diffusivity; CTh = cortical thickness; df = degrees of freedom; GENFI-CDR-SOB = GENFI Clinical Dementia Rating Sum-of-Boxes; LMEM = linear mixed-effects model

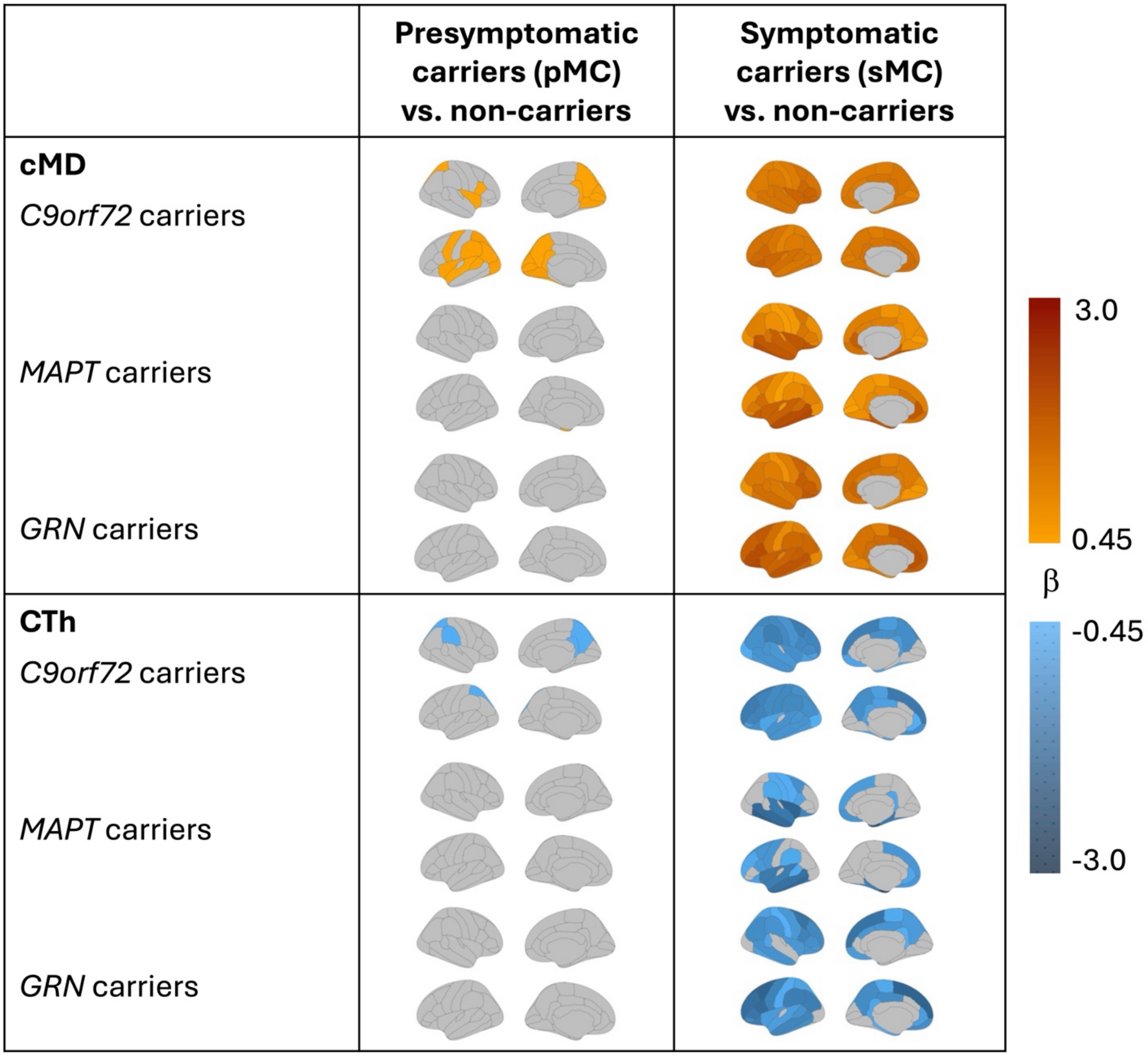

Supplementary Fig. 1 Brain maps illustrating the regional cMD and CTh topographical patterns in mutation carriers (presymptomatic [pMC] and symptomatic [sMC] mutation carriers) vs non-carriers as controls. The top panel displays β coefficients from linear mixed-effects models indicating increased cMD (red tones) in mutation carriers vs non-carriers, and the lower panel shows β coefficients indicating regions of reduced CTh (blue tones). The scale represents β values ranging from 0.45 (light orange) to 3.0 (dark red) corresponding to elevated cMD in mutation carriers vs non-carriers, and from -0.45 (light blue) to -3.0 (dark blue) corresponding to reduced CTh in mutation carriers vs non-carriers. All analyses in mutation carriers are presented stratified by mutation type (*C9orf72*, *GRN* and *MAPT*). Only regions with *P*-values adjusted for multiple comparisons < 0.05 (two-sided tests) are coloured. cMD = cortical mean diffusivity; CTh = cortical thickness; pMC = presymptomatic mutation carriers; sMC = symptomatic mutation carriers.

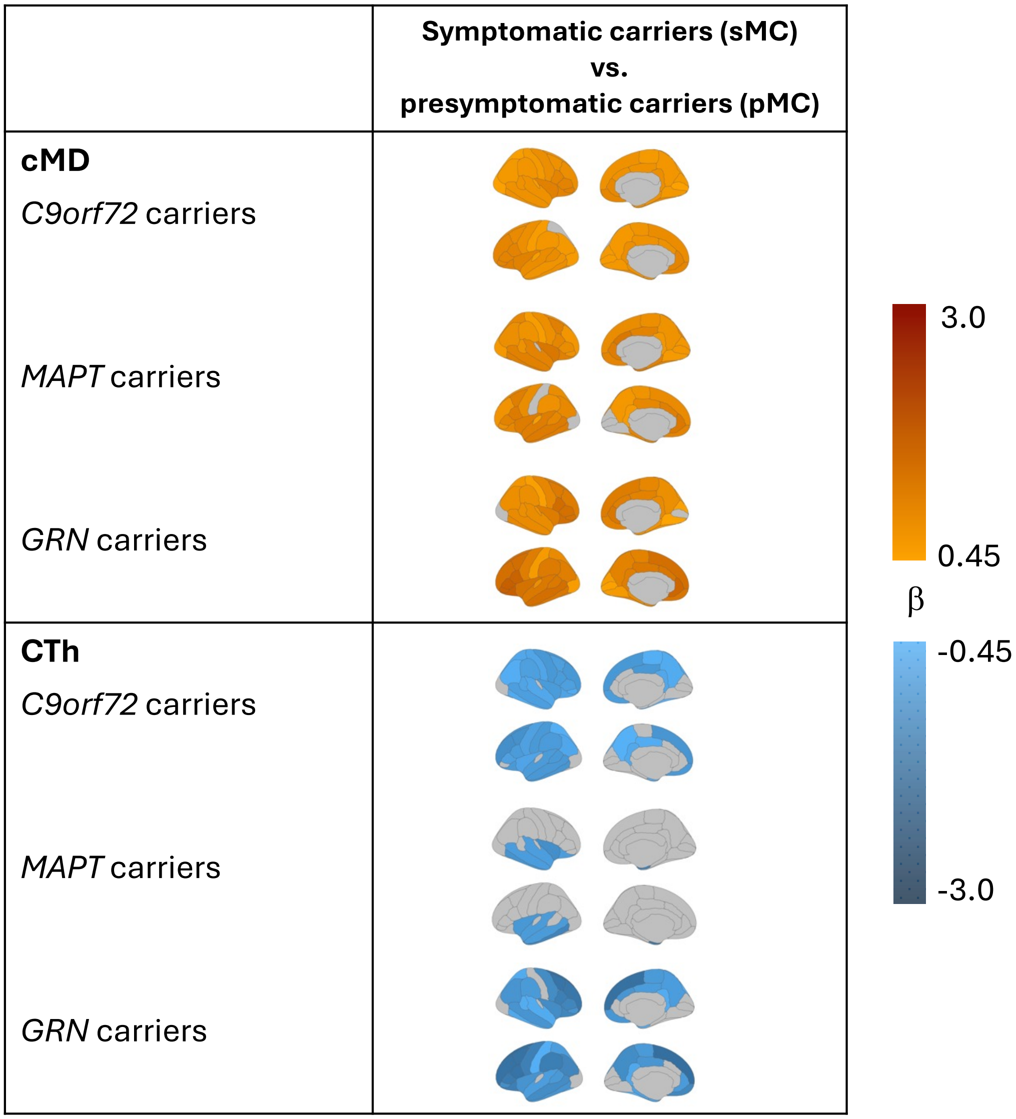

Supplementary Fig. 2 Brain maps illustrating the regional cMD and CTh topographical patterns in symptomatic (sMC) vs presymptomatic (pMC) mutation carriers. The top panel displays β coefficients from linear mixed-effects models indicating increased cMD (red tones) in sMC vs pMC, and the lower panel shows β coefficients indicating regions of reduced CTh (blue tones). The scale represents β values ranging from 0.45 (light orange) to 3.0 (dark red) corresponding to elevated cMD in mutation carriers vs non-carriers, and from -0.45 (light blue) to -3.0 (dark blue) corresponding to reduced CTh in mutation carriers vs non-carriers. All analyses in mutation carriers are presented stratified by mutation type (*C9orf72*, *GRN* and *MAPT*). Only regions with *P*-values adjusted for multiple comparisons < 0.05 (two-sided tests) are coloured. cMD = cortical mean diffusivity; CTh = cortical thickness; pMC = presymptomatic mutation carriers; sMC = symptomatic mutation carriers.

List of GENFI consortium co-investigators

Author	Affiliation
Rhian Convery	Department of Neurodegenerative Disease, Dementia Research Centre, UCL Queen Square Institute of Neurology, London, UK
Martina Bocchetta	Department of Neurodegenerative Disease, Dementia Research Centre, UCL Queen Square Institute of Neurology, London, UK
David Cash	Department of Neurodegenerative Disease, Dementia Research Centre, UCL Queen Square Institute of Neurology, London, UK
Sophie Goldsmith	Department of Neurodegenerative Disease, Dementia Research Centre, UCL Queen Square Institute of Neurology, London, UK
Kiran Samra	Department of Neurodegenerative Disease, Dementia Research Centre, UCL Queen Square Institute of Neurology, London, UK
David L. Thomas	Neuroimaging Analysis Centre, Department of Brain Repair and Rehabilitation, UCL Institute of Neurology, Queen Square, London, UK
Antonella Alberici	Centre for Neurodegenerative Disorders, Department of Clinical and Experimental Sciences, University of Brescia, Brescia, Italy
Enrico Premi	Stroke Unit, ASST Brescia Hospital, Brescia, Italy
Roberto Gasparotti	Neuroradiology Unit, University of Brescia, Brescia, Italy
Emanuele Buratti	ICGEB Trieste, Italy
Valentina Cantoni	Centre for Neurodegenerative Disorders, Department of Clinical and Experimental Sciences, University of Brescia, Brescia, Italy
Andrea Arighi	Fondazione IRCCS Ca' Granda Ospedale Maggiore Policlinico, Neurodegenerative Diseases Unit, Milan, Italy
Vittoria Borracci	Fondazione IRCCS Ca' Granda Ospedale Maggiore Policlinico, Neurodegenerative Diseases Unit, Milan, Italy
Maria Serpente	Fondazione IRCCS Ca' Granda Ospedale Maggiore Policlinico, Neurodegenerative Diseases Unit, Milan, Italy
Tiziana Carandini	Fondazione IRCCS Ca' Granda Ospedale Maggiore Policlinico, Neurodegenerative Diseases Unit, Milan, Italy
Emanuela Rotondo	Fondazione IRCCS Ca' Granda Ospedale Maggiore Policlinico, Neurodegenerative Diseases Unit, Milan, Italy
Chiara Fenoglio	Dept. of Biomedical, Surgical and Dental Sciences, University of Milan, Milan, Italy; Fondazione IRCCS Ca' Granda Ospedale Maggiore Policlinico, Neurodegenerative Diseases Unit, Milan, Italy
David Tang-Wai	The University Health Network, Krembil Research Institute, Toronto, Canada
Ekaterina Rogaeva	Tanz Centre for Research in Neurodegenerative Diseases, University of Toronto, Toronto, Canada
Miguel Castelo-Branco	Faculty of Medicine, ICNAS, CIBIT, University of Coimbra, Coimbra, Portugal.
Morris Freedman	Baycrest Health Sciences, Rotman Research Institute, University of Toronto, Toronto, Canada
Ron Keren	The University Health Network, Toronto Rehabilitation Institute, Toronto, Canada
Sandra Black	Sunnybrook Health Sciences Centre, Sunnybrook Research Institute, University of Toronto, Toronto, Canada

Sara Mitchell	Sunnybrook Health Sciences Centre, Sunnybrook Research Institute, University of Toronto, Toronto, Canada
Christen Shoesmith	Department of Clinical Neurological Sciences, University of Western Ontario, London, Ontario, Canada
Robart Bartha	Department of Medical Biophysics, The University of Western Ontario, London, Ontario, Canada; Centre for Functional and Metabolic Mapping, Robarts Research Institute, The University of Western Ontario, London, Ontario, Canada
Rosa Rademakers	Center for Molecular Neurology, University of Antwerp
Jackie Poos	Department of Neurology, Erasmus Medical Center, Rotterdam, Netherlands
Janne M. Papma	Department of Neurology, Erasmus Medical Center, Rotterdam, Netherlands
Lucia Giannini	Department of Neurology, Erasmus Medical Center, Rotterdam, Netherlands
Liset de Boer	Department of Neurology, Erasmus Medical Center, Rotterdam, Netherlands
Julie de Houwer	Department of Neurology, Erasmus Medical Center, Rotterdam, Netherlands
Rick van Minkelen	Department of Clinical Genetics, Erasmus Medical Center, Rotterdam, Netherlands
Yolande Pijnenburg	Amsterdam University Medical Centre, Amsterdam VUmc, Amsterdam, Netherlands
Mattias Nilsson	Department of Clinical Neuroscience, Karolinska Institutet, Stockholm, Sweden
Henrik Viklund	Karolinska University Hospital Huddinge
Tobias Langheinrich	Division of Neuroscience and Experimental Psychology, Wolfson Molecular Imaging Centre, University of Manchester, Manchester, UK; Manchester Centre for Clinical Neurosciences, Department of Neurology, Salford Royal NHS Foundation Trust, Manchester, UK
Albert Lladó	Alzheimer's disease and Other Cognitive Disorders Unit, Neurology Service, Hospital Clínic de Barcelona, Barcelona, Spain
Anna Antonell	Alzheimer's disease and Other Cognitive Disorders Unit, Neurology Service, Hospital Clínic de Barcelona, Barcelona, Spain
Jaume Olives	Alzheimer's disease and Other Cognitive Disorders Unit, Neurology Service, Hospital Clínic de Barcelona, Barcelona, Spain
Mircea Balasa	Alzheimer's disease and Other Cognitive Disorders Unit, Neurology Service, Hospital Clínic de Barcelona, Barcelona, Spain
Nuria Bargalló	Imaging Diagnostic Center, Hospital Clínic de Barcelona, Barcelona, Spain
Sergi Borrego-Ecija	Alzheimer's disease and Other Cognitive Disorders Unit, Neurology Service, Hospital Clínic de Barcelona, Barcelona, Spain
Alazne Gabilondo	Cognitive Disorders Unit, Department of Neurology, Donostia University Hospital, San Sebastian, Gipuzkoa, Spain; Neuroscience Area, Biodonostia Health Research Insitute, San Sebastian, Gipuzkoa, Spain
Ioana Croitoru	Neuroscience Area, Biodonostia Health Research Insitute, San Sebastian, Gipuzkoa, Spain
Mikel Tainta	Neuroscience Area, Biodonostia Health Research Insitute, San Sebastian, Gipuzkoa, Spain
Myriam Barandiaran	Cognitive Disorders Unit, Department of Neurology, Donostia University Hospital, San Sebastian, Gipuzkoa, Spain; Neuroscience Area, Biodonostia Health Research Insitute, San Sebastian, Gipuzkoa, Spain
Patricia Alves	Neuroscience Area, Biodonostia Health Research Insitute, San Sebastian, Gipuzkoa, Spain; Department of Educational Psychology and

	Psychobiology, Faculty of Education, International University of La Rioja, Logroño, Spain
Benjamin Bender	Department of Diagnostic and Interventional Neuroradiology, University of Tübingen, Tübingen, Germany
David Mengel	Department of Neurodegenerative Diseases, Hertie-Institute for Clinical Brain Research and Center of Neurology, University of Tübingen, Tübingen, Germany; Center for Neurodegenerative Diseases (DZNE), Tübingen, Germany
Lisa Graf	Department of Neurodegenerative Diseases, Hertie-Institute for Clinical Brain Research and Center of Neurology, University of Tübingen, Tübingen, Germany
Annick Vogels	Department of Human Genetics, KU Leuven, Leuven, Belgium
Mathieu Vandenbulcke	Geriatric Psychiatry Service, University Hospitals Leuven, Belgium; Neuropsychiatry, Department of Neurosciences, KU Leuven, Leuven, Belgium
Philip Van Damme	Neurology Service, University Hospitals Leuven, Belgium; Laboratory for Neurobiology, VIB-KU Leuven Centre for Brain Research, Leuven, Belgium
Koen Poesen	Laboratory for Molecular Neurobiomarker Research, KU Leuven, Leuven, Belgium
Pedro Rosa-Neto	Translational Neuroimaging Laboratory, McGill Centre for Studies in Aging, McGill University, Montreal, Québec, Canada
Maxime Montembault	Douglas Research Centre, Department of Psychiatry, McGill University, Montreal, Québec, Canada
